# Initial Evidence That Gilthead Seabream (*Sparus aurata* L.) Is a Host for Lymphocystis Disease Virus Genotype I

**DOI:** 10.3390/ani11113032

**Published:** 2021-10-22

**Authors:** Mohamed Shawky, Engy Taha, Basem Ahmed, Mahmoud Aly Mahmoud, Mohamed Abdelaziz, Mohamed Faisal, Ausama Yousif

**Affiliations:** 1 Department of Virology, Faculty of Veterinary Medicine, Cairo University, Giza 12211, Egypt; dr.shawky92@gmail.com (M.S.); basem-ahmed@cu.edu.eg (B.A.); 2 Department of Aquatic Animal Medicine and Management, Faculty of Veterinary Medicine, Cairo University, Giza 12211, Egypt; engytahavet9@gmail.com (E.T.); mabdelaziz1973@yahoo.com (M.A.); 3 Department of Pathology, Faculty of Veterinary Medicine, Cairo University, Giza 12211, Egypt; mahmoudaly@cu.edu.eg; 4 Department of Pathobiology and Diagnostic Investigation, College of Veterinary Medicine, Michigan State University, 1129 Farm Lane, Room 340G, East Lansing, MI 48824, USA; faisal@cvm.msu.edu; 5 Faculty of Veterinary Medicine, Benha University, Moshtohor, Tukh 13736, Egypt

**Keywords:** lymphocystis disease virus, LCDV genotype I, major capsid protein, gilthead seabream, histopathology, phylogenetic analysis

## Abstract

**Simple Summary:**

Nodular lesions were observed on the skin and fins of 95% of one and a half million juvenile gilthead seabreams cultured in Egypt, shortly after importation from Europe. We undertook a study to describe the clinical disease course, identify the causative agent, and investigate the origin of the causative agent. Preliminary diagnosis based on gross lesions and postmortem examination suggested lymphocystis disease caused by lymphocystis disease virus, *Lymphocystivirus, Iridoviridae.* Histopathological and ultrastructural pictures were typical of lymphocystis disease virus infections. Polymerase Chain Reaction followed by sequencing and phylogenetic analysis of the major capsid protein gene demonstrated the presence of lymphocystis disease virus genotype I, originally associated with lymphocystis disease in Northern European countries, with 99.7% and 100% nucleotide and deduced amino acid identity values, respectively. Lymphocystis disease virus genotype I has never been reported in this species or in the region. Regardless of whether it has maintained a previously undetected state of endemicity in Egypt or was introduced through importation or contamination of ship ballast water, the findings of this study add to existing knowledge about the lymphocystis disease’s ecology, and lymphocystis disease virus genotypes and their host range.

**Abstract:**

Marine and brackish water aquacultures are rapidly expanding in the Mediterranean basin. In this context, Egypt recently received a shipment of a 1.5 million juvenile gilthead seabream (*Sparus aurata* L.) from European Mediterranean facility. Within a few weeks of their arrival, 95% of the imported fish developed nodules on their skin and fins that lasted for several months. This study was undertaken to describe the clinical disease course, to identify the causative agent, and to investigate its origin. Preliminary diagnosis based on gross lesions and postmortem examination suggested lymphocystis disease (LCD), caused by the lymphocystis disease virus (LCDV; genus *Lymphocystivirus*, family *Iridoviridae*). Histopathological and ultrastructural features were typical of LCDV infections. PCR followed by sequencing and phylogenetic analysis of a 306-bp fragment of the major capsid protein (MCP) gene demonstrated the presence of LCDV genotype I, originally associated with LCD in Northern European countries, with 99.7% and 100% nucleotide and deduced amino acid identity values, respectively. LCDV genotype I has neither been reported in this species nor in the region. Regardless of the source of infection, findings of this study add to existing knowledge about the ecology of LCDV genotype I and its host range.

## 1. Introduction

The ever-increasing demand for high-value protein in Egypt led to an expansion in aquaculture over the last four decades [1]. Today, Egypt is ranked ninth worldwide and first in Africa in fish production from aquaculture [2]. In Egypt, like in other marine aquaculture activities in the Mediterranean region, special attention has been given to the highly prized fish, gilthead seabream (*Sparus aurata* L. Pisces: *Sparidae*). The euryhaline and eurythermal habits of this species [3] made it a perfect fit for the range of salinities and temperature prevalent in Egypt. Unfortunately, this rapid development of gilthead seabream farming has not been accompanied by the development of an effective health management plan to minimize the potential risks to this growing industry from introduced or endemic pathogens.

A common practice in aquaculture is the exchange of live fish or their gametes in search of better growth and disease resistance traits [4]. There are several well-documented cases of unintentional pathogen transmission through international trade of live fish and their products [4]. Several highly pathogenic viruses were introduced into negative zones through carrier fish and infected eggs, such as infectious hematopoietic necrosis virus [5], koi herpesvirus [6], pilchard herpesvirus [7], and salmonid alphavirus (sleeping disease) [8]. Several private fish growers and governmental agencies in Egypt have imported live fish of several species from different parts of the world as a seed for new aquaculture projects. These shipments were accompanied by health inspection reports certifying their freedom of reportable fish diseases as stipulated by the World Animal Health Organization [9]. Despite the certification, many non-reportable yet serious pathogens can be introduced due to the severe gap of current knowledge on diseases of marine fishes, or absence of specific and sensitive diagnostic assays [4].

One of the examples of viruses that were disseminated throughout the Atlantic coasts of Europe and the Mediterranean along with the international trade of gilthead seabream is the Lymphocystis Disease Virus (LCDV, genus *Lymphocystivirus*, family *Iridoviridae*), the causative agent of Lymphocystis Disease (LCD) [10]. LCD is characterized by the formation of numerous nodules, each made of a single hypertrophied cell that often coalesce forming a tumor-like mass on skin and fins, and rarely on internal organs [11]. Although mortality seldom occurs, LCD can cause significant economic losses related to non-marketability, secondary infections, and poor growth rate of fish [10]. LCDV is a double-stranded DNA, icosahedral virus, measuring up to 350 nm in diameter, and has a very wide host range of marine and freshwater fish species [10]. Like in other iridoviruses, the major capsid protein (MCP) is the main viral protein structure, and the complete or partial sequence of its gene has been widely used in phylogenetic studies [10,12]. Currently, LCDV isolates have been grouped into nine genotypes with all known isolates originating from *S. aurata* clustering in genotype VII [13,14].

Recently, Egypt received a health-certified shipment of one million and a half juvenile *S. aurata* from European Mediterranean facility. Within a few weeks of their arrival, 95% of the imported fish developed nodules on their skin and fins that lasted for several months. This study was undertaken to describe the clinical disease course, to identify the causative agent, and to discuss its potential origin.

## 2. Materials and Methods

### 2.1. Fish and Sampling

In 2016, 1.5 million juvenile gilthead seabreams were imported from a Mediterranean mariculture facility to a mariculture facility in Egypt. Upon arrival, the 14-week-old fish (5.0 g + 0.26 g in weight and 6.6 cm + 0.42 cm in total length) were stocked equally in 20 (1-acre) earthen ponds. The ponds received water from the Suez Canal. The fish were fed on 1.5 mm artificial dry feed (Aller Aqua Egypt Co., Giza, Egypt). Water flow was adjusted to 2.25 m^3^/ min. In ponds, salinity was 37 + 3.3 ppt, oxygen level was 4.76 + 0.7 mg/L. and water temperature ranged from 15.5 °C + 0.21 °C in December to 14 °C + 1.0 °C in January and February 2017, then rose gradually to reach 19.0 °C + 2.27 °C in April. No other fish species were stocked in these ponds. Every 2–3 weeks, a subsample (700–1000 fish) of the farmed fish was collected by nets, their weights were recorded, and they were visually examined for health signs.

Following 25 days of pond stocking, minute grayish white nodules were observed infrequently on a few fish in each pond, first on the fins, especially the caudal fin, then the skin on both sides of affected fish. In the following two weeks, the number of affected fish rose dramatically to reach up to 95 + 0.8% in all ponds during the months of January and February 2017. The morbidity rate decreased dramatically as the water temperature increased, dropping to 40% in March 2017 and then further in April (5%) until it could not be observed in May and subsequent summer months. Despite the high morbidity rate, mortality was seldom observed. No lesions or cysts were observed in the internal organs of any of the fish examined.

From each pond, representative fish were collected, weight and length were recorded, and they were humanely euthanized with an overdose of tricaine methane sulfonate (MS-222, Sigma Aldrich, St. Louis, MI, USA). All handling and sampling of fish were performed in accordance with the guidelines of the Institutional Animal Care and Use Committee of Cairo University, Egypt (Approval number CU-II-F-24-19).

### 2.2. Histopathology

Cysts and surrounding tissues from the skin and fins, as well as random samples from the gills and internal organs (liver, spleen, and kidney) were collected and then fixed in 10% buffered formalin. Tissues were then dehydrated in ethanol, cleared in xylene, and embedded into paraffin wax. Standard histological protocols and sectioning (5–10 µm) were performed on slides and stained with hematoxylin and eosin (H&E) [15,16].

### 2.3. Electron Microscopy

Portions of lesions on the caudal fin and body flanks were cut into ~1 mm sections. Sliced tissues were processed for transmission electron microscopy by fixation in 2.5% glutaraldehyde (Sigma Aldrich) in phosphate buffered saline (PBS; 0.1 mol/L, pH 7.4) for 2 h at 4 °C, postfixed in 1% osmium tetroxide (Sigma Aldrich) in PBS for 1 h at 4 °C, dehydrated in alcohol, and embedded in an epoxy resin. Microtome sections were prepared at approximately 500–1000 µm thickness with a Leica Ultra cut UCT microtome (Leica, Wetzlar, Germany). Semi-thin sections were stained with 1% toluidine blue (Sigma) and then examined by HD camera Leica ICC50. Ultra-thin sections were prepared further at approximately 30–100 nm thickness and were stained with uranyl acetate and lead citrate. Sections were examined by using a JEOL JEM-1400 TEM (JEOL, Peabody, Massachusetts, USA) at the Transmission Electron Microscopy Laboratory, Faculty of Agriculture, Cairo University Research Park.

### 2.4. Molecular Analysis

Molecular analysis was performed due to the suspicion, based upon gross observations, that LCDV was associated with the observed morbidity. DNA was extracted from nodules taken from fins and skin using GF-1 Tissue Blood Combi DNA Extraction Kit (Vivantis, Malaysia). DNA quality was assessed by electrophoresis in 0.8% agarose gel and ethidium bromide staining. PCR was performed in GeneAmp^®^ PCR System 9700 thermal cycler (Thermo Fisher Scientific, Bremen, Germany), using the DreamTaq Green PCR Master Mix (2X) (Thermo Scientific™, Bremen, Germany) in a total of 25 μL reaction volume. Oligonucleotide primers targeting the MCP gene of LCDV were used as detailed in [12]. Each reaction contained 0.25 μM of each primer and 30 ng of DNA. Cycling parameters were: 30 s at 95 °C, 30 s at 54 °C, and 1 min at 72 °C for 40 cycles [12]. PCR product was analyzed in 1.5% agarose gel containing ethidium bromide and visualized under U.V. light, and the resultant amplicon was sent to the Colors Laboratory, Cairo, Egypt for PCR purification and sequencing.

Obtained sequences were compared to other LCDV sequences originating from different regions of the world (Table 1) using NCBI nucleotide BLAST^®^ tool [17]. In brief, all sequences were aligned using ClustalW program embedded in BoiEdit^®^ 7.0.5.3. Initial tree(s) for the heuristic search were obtained automatically by applying Neighbor-Join and BioNJ algorithms to a matrix of pairwise distances estimated using the Maximum Composite Likelihood (MCL) approach and then selecting the topology with superior log likelihood value. The tree was drawn to scale, with branch lengths measured in the number of substitutions per site. Phylogenetic analyses were conducted using MEGA X software [18]. Distances between amino acid sequences of LCDV-SA_EG MCP sequence and other LCDV genotypes were calculated using MegAlign© software of LaserGene package version 7.

## 3. Results

### 3.1. Clinical Observations

**Figure 1:**
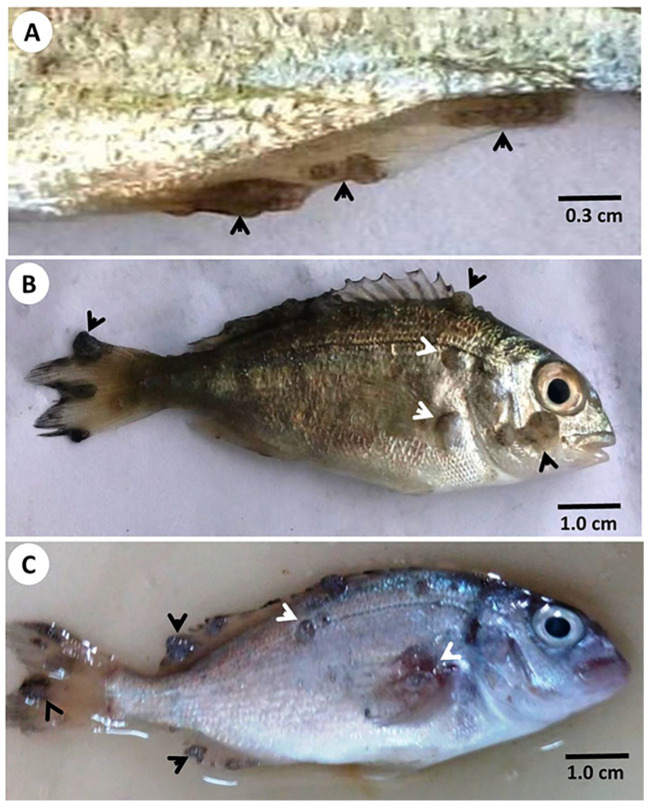
Gross external lesions on affected gilthead seabream (*Sparus aurata*) (arrowheads). (**A**): Early lesions on the pelvic fin (arrowheads). (**B**): Larger lesions with well-demarcated nodular formation on lateral and ventral surface of the fish (arrowheads). (**C**): Coalesced lesions scattered all over the fish surface (arrowheads).

Both gross external clinical signs—the grayish white nodules measuring 1–3 mm in diameter, which often coalesced to form a larger lesion surrounded with necrotic and hemorrhagic zones (Figure 1)—and postmortem examination led to the suspicion that the gilthead seabream of this study suffered from lymphocystis disease caused by LCDV. The nodules appeared first at the tips of fins, then increased in size progressively, some of which coalesced together forming a bigger mass. Sloughed nodules left no scars or gross signs of tissue reaction. No nodules were grossly observed on or in the internal organs.

### 3.2. Histopathological Findings

**Figure 2:**
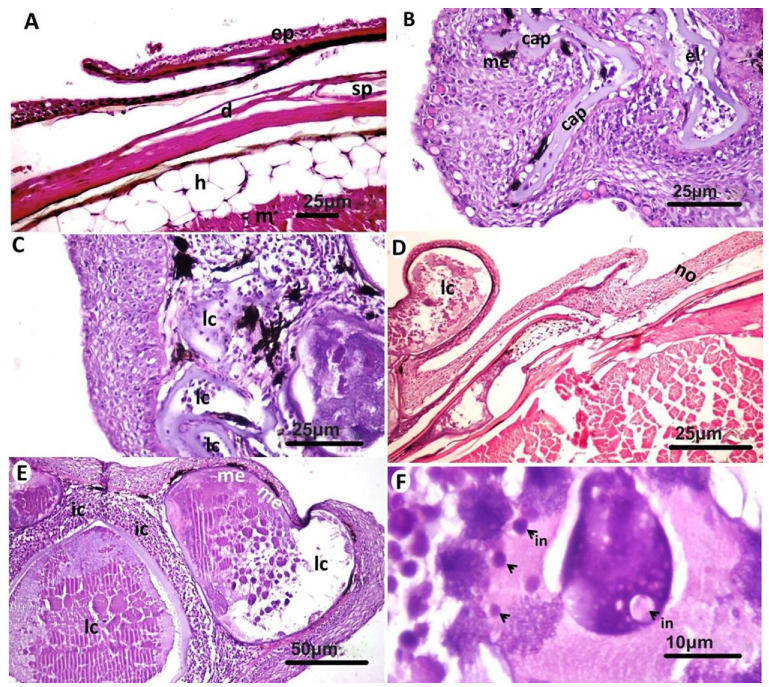
Microscopic pathological examination of gilthead seabream (*Sparus aurata*) skin. (**A**): Apparently normal skin area with normal epidermis (ep), dermis (d), scale pocket (sp), hypodermis (h), and muscle layer (m). (**B**): Early lymphocystis lesion (el) containing small numbers of fibroblastic cells and surrounded with thick basophilic capsule (cap) and melanophores (me). (**C**): More enlarged lymphocystis lesion (lc) with thin capsule containing many fibroblastic cells. (**D**): Severely enlarged lymphocystis lesion (lc) protruded over the skin, while other parts of the skin were apparently normal (no). (**E**): Lymphocystis cells (lc) containing basophilic tissue debris and edematous fluid without prominent fibroblastic cells. The cysts were surrounded by inflammatory cells (ic). (**F**): Basophilic and eosinophilic inclusion bodies (in) in lymphocystis cells. (H&E).

**Figure 3:**
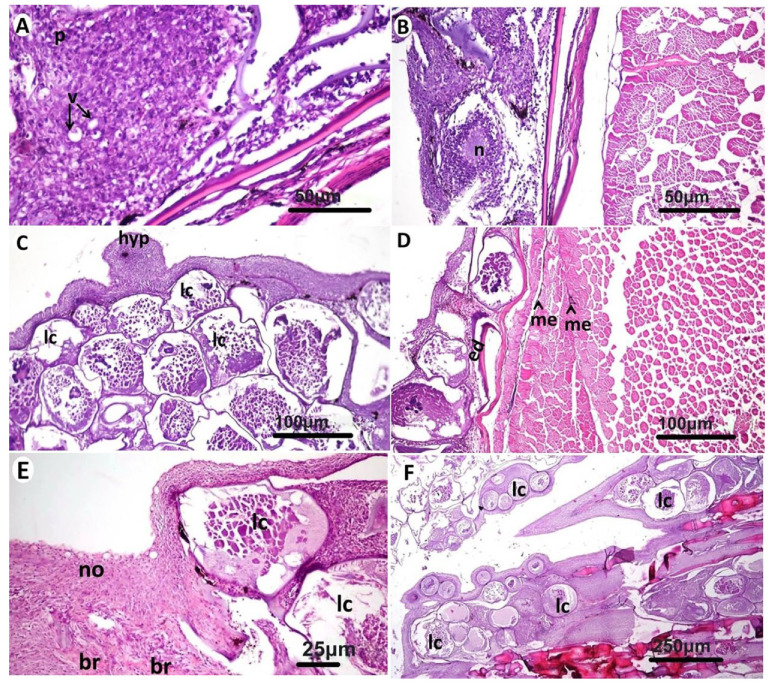
Microscopic pathological examination of affected gilthead seabream (*Sparus aurata*) fins. (**A**): Epidermal layer showing vacuolar degeneration (v) and pyknotic nuclei (p). (**B**): Melanin pigment aggregation and necrosis (n) in the epidermis. (**C**): Nodular epidermal hyperplastic proliferation (hyp) and multiple lymphocystis cells (lc). (**D**): Edema in scale pocket (ed) and few melanophores aggregated (me) in the underlying musculature. (**E**): Lymphocystis cells (lc) in fins adjacent to bony rays (br) beside apparently normal (no) fin tissue. (**F**): Multiple lymphocystis cells (lc) of variable sizes in the fins. (H&E).

Microscopic examination of affected gilthead seabream skin (Figure 2A) revealed the presence of a layer that was formed of multiple subepidermal cyst-like lesions that often extended to occupy the whole dermis layer. The cystic lesions appeared in different stages of development with the presence of thick basophilic hyaline capsule at the early stages surrounding a group of fibroblastic cells and margined outside with melanophores (Figure 2B). As the lesion advanced, the cysts appeared more enlarged, and the capsule appeared as a thin layer separating the cysts and contained more fibroblastic cells (Figure 2C). Some of the cysts were severely enlarged and protruded over the skin (Figure 2D). In advanced stages, the cysts contained basophilic tissue debris and edematous fluid but no prominent individual fibroblast cells. At this stage, the infiltration of mononuclear cells was noticed around and between the cysts (Figure 2E), but the predominant type of the cells were lymphocytes. The lymphocystis cells at this stage contained multiple basophilic and acidophilic inclusion bodies (Figure 2F). In the epidermis, vacuolar degeneration and necrotic areas were common (Figure 3A,B), and multiple melanophores were observed nearby the necrotic tissue. Nodular hyperplasia was a common finding in the epidermis (Figure 3C). Other layers of the skin were apparently normal except mild edema in scale pockets together with some melanophores aggregated in the underlying musculature (Figure 3D). The same lesions were observed in the fins, but in some areas, the lymphocystis cells were few and appeared beside the normal tissue (Figure 3E), while in other fin regions, several lymphocystis cells were observed in the soft fin tissue or between the bony rays (Figure 3F).

**Figure 4:**
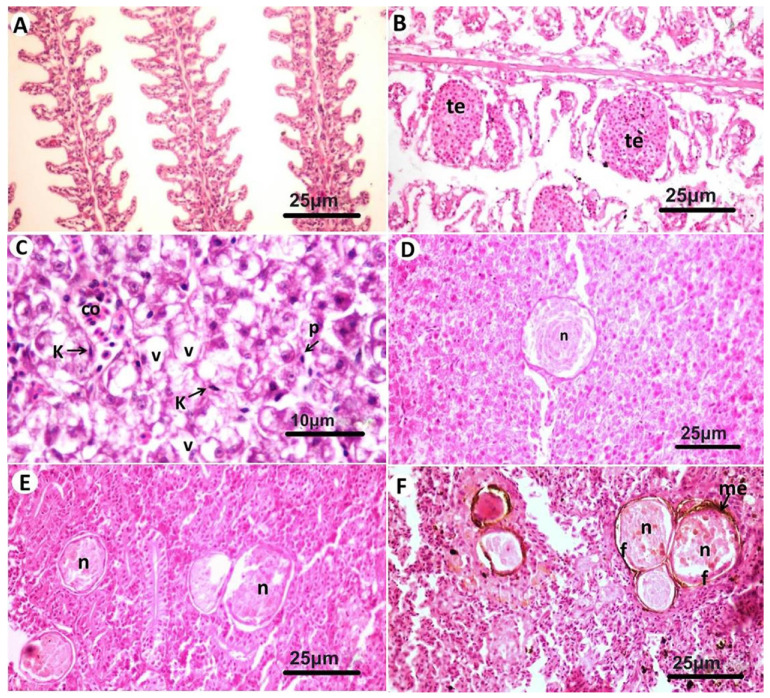
Microscopic examination of gills and internal organs of gilthead seabream (*Sparus aurata*). (**A**): Apparently normal branchial tissue. (**B**): Lamellar telangiectasis (te) in gills. (**C**): Congestion (co) of hepatic sinusoids, vacuolar degeneration (v) of hepatocytes, and Kupffer cells proliferation (k). (**D**): Focal necrotic areas (n) along the hepatic tissue surrounded with proliferated fibroblasts (f). (**E**) Multiple necrotic foci (n) in the renal tissue. (**F**) Necrotic foci (n) in spleen surrounded by melano-macrophage centers (me) and fibroblasts (f). (H&E).

Various microscopic lesions were observed in gills, liver, kidneys, and spleen. Lamellar telangiectasis was common in gills (Figure 4A,B). Congestion of sinusoidal capillaries, vacuolar degeneration of hepatocytes, Kupffer cells proliferation, and pyknosis of some hepatocytes’ nuclei were observed in the liver (Figure 4C). In a few cases, focal necrotic areas were noticed in the hepatic tissue (Figure 4D). Necrotic tissues in kidneys and spleens showed prominent melano-macrophage centers especially in the spleen (Figure 4E,F).

### 3.3. Electron Microscopy

**Figure 5:**
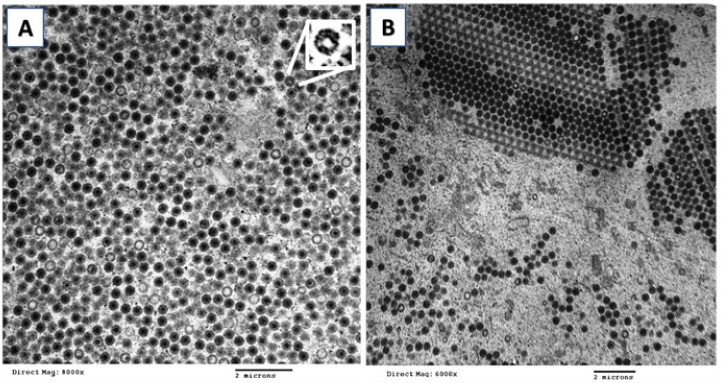
Transmission electron microscope images of ultra-thin sections of lymphocystis lesions on skin and fins of gilthead seabream (*Sparus aurata*). (**A**) Scattered large icosahedral particles in different stages of morphogenesis (8000×). Inset shows empty double-layered capsids. (**B**) Crystalloid structure with densely packed virus particles measuring about 350 nm with a dense core (6000×).

Examination of lymphocystis cells of gilthead seabream under TEM revealed numerous electron dense hexagonal viral particles in the cytoplasm. The scattered particles were present in an electron lucent background with multiple developmental stages, from empty particles to particles containing an electron-dense core (Figure 5A).

Assembled particles appeared in para-crystalline arrays in the cytoplasm with a low number of empty particles (Figure 5B). Hexagonal mature viral particles were about 350 nm in diameter with a concentric core about 150 nm.

### 3.4. Gene Sequencing and Phylogenetic Analysis

PCR amplification yielded a 306 bp product (Figure S1) that is consistent with partial amplification of the MCP gene of LCDV [12]. BLAST analysis revealed that the study sequence designated LCDV-SA_EG (Genbank Acc. MN128712) was 99.65% identical to the reference LCDV1, belonging to genotype I, sequence (Genbank Acc.L63545.1; Table 1 and Table S1; Figures S1 and S3).

Based on nucleotide sequences, representatives of genotypes III, IX, VI, VIII, IV, VII, V, and II were 83.3, 82.9, 82.3, 81, 79.4, 78.9, 78%, and 77.2% identical to LCDV-SA_EG, respectively. Where multiple representatives of the same genotype were analyzed, differences in identity percentages were generally below 1%, and only the highest identities were reported above.

The deduced amino acid sequence was identical to that of the reference LCDV1 (Table 2). However, the study sequence was divergent from representatives of the other genotypes (divergence ranging from 10–15%; Table 2). Interestingly, the amino acid sequence divergence with the genotype previously reported to infect gilthead seabream (genotype VII) was 12.5%.

**Figure 6:**
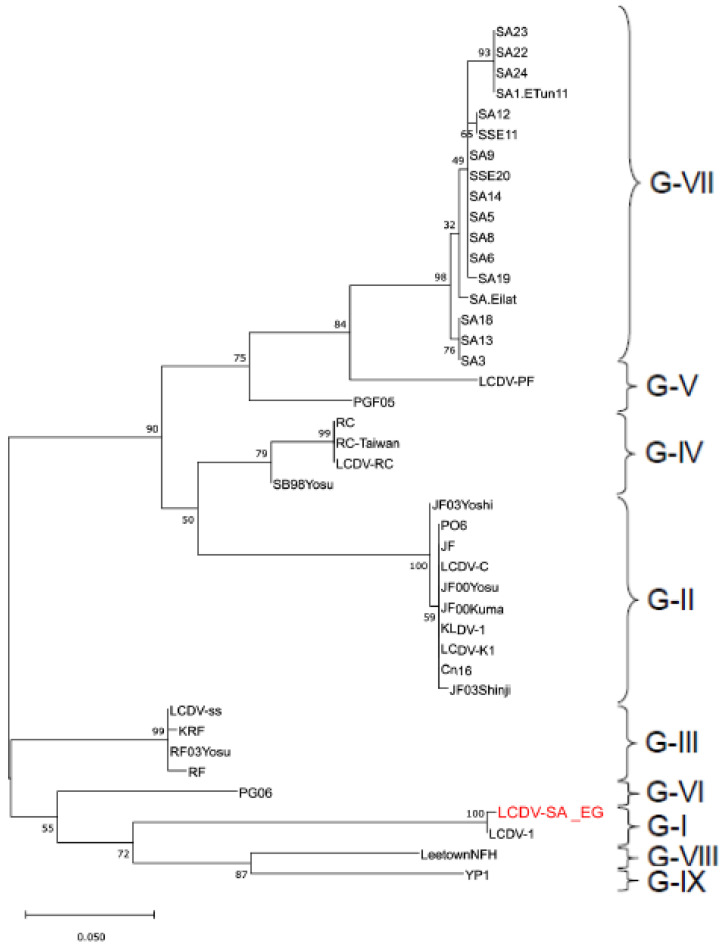
Dendrogram depicting the relationship between LCDV-SA_EG MCP sequence and representative sequences from other LCDV genotypes. The Maximum Likelihood method and Tamura−Nei model were used for phylogenetic analysis. The study sequence is in bold red. The percentage of trees in which the associated taxa are clustered together is shown next to the branches. The scale represents the number of substitutions per site.

Phylogenetic analysis using the Maximum Likelihood method clustered LCDV MCP gene sequences in a manner like that which has been previously published [13,14] and supported the division of isolates into genotypes. The study sequence was clustered with LCDV1 sequence in genotype I (Figure 6).

## 4. Discussion

Gross disease signs, heavy involvement of the skin and fins, microscopical tissue alterations, ultrastructural virus morphology, and phylogenetic studies proved that the gilthead seabream specimens from this study were infected with LCDV. This is not surprising since LCDV is known to have the widest host range of any known fish virus [11] and has been previously reported from two marine fish species from Alexandria, Egypt [19,20]. A series of reports from Southern Europe and Northern Africa indicate that LCDV is the most frequently detected virus in farmed *S. aurata* in the Mediterranean Sea and South Atlantic Ocean in countries such as Tunisia [21], Portugal [22], Spain [23], France [24], Greece, and Turkey [25]. The LCD episode described in this study was characterized by a morbidity rate that involved almost all fish and all ponds from the same imported lot, but not in neighboring ponds whose gilthead seabream originated from local sources, thereby suggesting that the importation, directly or indirectly, played a major role in this disease outbreak. Despite the striking similarity in clinical signs, the disease course of the current LCD outbreak was different from the first LCD outbreak ever described in cultured juvenile gilthead seabream in the nearby Gulf of Aqaba (Red Sea) by Paperna and his colleagues [26] in that the disease erupted within a week after the fish transfer into offshore marine cages in the Gulf and subsided within three weeks. The outbreak described herein lasted over three months with a peak morbidity of 95% that started to subside in the fourth month as the water temperature started to rise. This difference may be attributed to several factors, such as differences in water temperature and salinity, depth of the water column (shallow pond vs. offshore cages), the frequency of water changes and subsequent virus concentration (less virus load is expected for offshore cages), or the LCDV genotype involved.

The full genomes of three LCDV isolates have been published to date [10]. The LCDV genome is circularly permuted and terminally redundant dsDNA containing >150 open reading frames (ORFs). Isolates from different parts of the world differ in genome size, genome organization, and gene product identity. Because the MCP gene sequence is relatively conserved among members of the family *Iridoviridae*, its partial or full sequence has been widely used to perform phylogenetic studies [27]. When all available MCP nucleotide sequences of LCDV isolates from a variety of fish hosts and geographic regions were analyzed, the phylogenetic analyses proposed the presence of nine genotypes within the genus *Lymphocystivirus* [13,14]. Interestingly, so far, each genotype is limited to one or a few fish species [13,14,28], with all sequences of LCDV retrieved from gilthead seabream (*Sparus aurata*) and Senegalese sole (*Solea senegalensis*) clustering together to form genotype VII [13,21].

Unexpectedly, the LCDV partial MCP sequence obtained in this study, designated SA_EG, showed high similarity to comparable sequences of the LCDV genotype I isolates that infect the European flounder (*Platichtys flesus*) and plaice (*Pleuronectes platessa*) in Northern Europe [10]. On the contrary, the MCP sequence of LCDV-SA_EG was distinct from all other gilthead seabream sequences in genotype VII retrieved from the South Atlantic, Mediterranean Sea (i.e., Southern Europe), and Gulf of Aqaba. To the best of our knowledge, this is the first report to suggest that LCDV genotype I infects gilthead seabream or any fish species in the Mediterranean basin. The use of only partial MCP gene sequences and our inability to conduct experimental infection studies due to logistic and regulatory constraints prevented us from pinpointing host and/or virus factors that played a role in the establishment of LCDV genotype I infection in this new host species and geographic region.

Isolates of LCDV genotype I and VII also exhibit phenotypic differences beyond host range. For example, when Alonso and his colleagues [29] inoculated representative isolates of both genotypes on fish fibroblastic cells in vitro, virus titers and the time necessary to develop cytopathic effects (CPE) in infected cells varied between the *S. aurata* genotype VII isolates and those from Northern Europe. In the same context, antibodies developed against a gilthead seabream LCDV isolate did not react with any of the North European isolates, suggesting the presence of antigenic differences.

The differences between LCDV genotypes extend to in vivo infection. When Kitamura and his colleagues [28] attempted to experimentally infect fish from different families with various LCDV genotypes, the fish were infected only by the respective homologous isolate and not vice versa. This has led scientists to believe that each of the LCDV genotypes can only infect the fish species from which it originated. That was refuted by the work of Cano and her colleagues [13], who exhibited the cross-species transmission between gilthead seabream and Senegalese sole, belonging to two different fish families, with LCDV genotype VII isolates originating from one another.

The authors attributed the cross infection to the coexistence of the two fish species in marine aquaculture facilities for decades, which allowed the adaptation of the virus to other fish species. Findings of these studies on fish susceptibility to each of the LCDV genotypes raised a couple of questions: Where did LCDV genotype I infecting gilthead seabream in Egypt come from? Moreover, if genotype I is pathogenic to *S. aurata*, why was LCD not noticed in other ponds whose sea bream originated from Egyptian nurseries? An additional aim of this study was to determine if there are differences in the virus-induced histopathology in the imported seabream infected by genotype I vs. those described previously for the same species by genotype VII.

To answer the first question, one would first assume that LCDV genotype I came along with the imported gilthead seabream from endemic LCDV areas. While this explanation is plausible, a series of studies demonstrated that gilthead seabream in the Eastern Atlantic, Mediterranean, and Gulf of Aqaba mariculture are infected with LCDV-genotype VII isolates exclusively [13,21,30]. Therefore, it is highly unlikely that LCDV genotype I came in the fish shipment. Besides, the health certification that accompanied the imported live fish shipment showed no previous LCD history in the farm of origin. A second assumption is that LCDV genotype I may have been endemic in Egypt, although this genotype has not been reported from Egypt or any other country in the Middle East before. The fact that seabream of Egyptian origin farmed in the same facility did not develop LCD gives this explanation some credibility. Alternatively, that imported but not local seabream showed clinical disease is probably related to the importation-related stress which is known to increase fish susceptibility to infection [31].

The observation of the relatively narrow host range of each of LCDV genotype led Yan and his colleagues [32] to analyze the genetic diversity of LCDV and its evolutionary relationship with the respective fish hosts. The study included 25 LCDV isolates representing seven genotypes (I-VII) and their respective hosts. Yan and his colleagues [32] demonstrated that LCDV genotype I was the earliest to differentiate among all other LCDV genotypes, while genotype VII was the latest. Interestingly, when the phylogenies of the host fish species were compared to those of LCDV genotypes, no significant evidence of cospeciating between LCDVs and their host fish were found. As such, one can assume that at a certain time, LCDV1 was the only LCDV present on the planet, a matter that gives this genotype the time needed to adapt to multiple fish species and geographic areas. This may explain the existence of this genotype in Egypt. As phylogenetic studies expand to previously unstudied geographic areas, additional hosts to LCDV genotype I may be recognized.

A third possibility is that LCDV genotype I reached the farm in Egypt through the main water source originating from the Suez Canal, a major waterway that connects the Mediterranean and Red seas with heavy traffic of freighters and oil tankers. It is possible that the virus was brought to Egypt from Northern Europe through the ballast water that these ships discharge. Indeed, there is a growing concern that ballast water plays an important role in intercontinental pathogen transmission [4]. Last, although brine shrimp (*Artemia spp*., *Crustacea, Branchiopoda, Anostraca*), a major food source in aquaculture for fish larval stages, is produced locally and commercialized, many fish growers import brine shrimp cysts from Europe and North America. A study by Valverde and his colleagues [33] demonstrated that LCDV can be transmitted by *Artemia* cysts and metanauplii, and the infectivity of the *Artemia* LCDV to fish was confirmed. A further study by Cano and her colleagues [34] demonstrated that LCDV infectious particles persist in *Artemia* through its entire life cycle, suggesting that *Artemia* might act as a reservoir of LCDV. It is, therefore, possible that *Artemia* cysts imported from LCDV-1 endemic areas introduced this genotype to Egypt. Regardless of whether LCDV genotype I is endemic to Egypt or had been introduced, findings of this study add to the existing knowledge about the ecology of LCD and LCDV genotypes.

The histopathological changes noticed in affected gilthead seabream were very much like those described before for this species in Southern Europe [35,36]. Lesions were observed not only in skin and fins but also in internal organs. The LCD classical hypertrophied lymphocystis cells were restricted in distribution to both the dermis and sub dermis and never found in the hypodermis, muscular tissue, or internal organs. Each nodule consisted of one or more lymphocystis cells, and LCDV particles could be visualized in these cells, indicating its involvement in lymphocystis cell formation. Similar findings were reported earlier on the ultrastructural virus morphology in *S. aurata* lymphocystis cells [19,24] and histopathology [36,37]. On the contrary, lymphocystis nodules were observed on the mesentery and internal organs of other fish species [11]. In this study, several lesions were found in internal organs despite the absence of nodules, confirming the systemic nature of LCD in *S. aurata*. When Cano and his colleagues [35] applied immunohistochemistry and in situ hybridization to determine the affected tissues in LCDV-infected *S. aurata*, they found a widespread distribution of the virus antigens and DNA in the lesions, even in areas without any tissue alterations in internal organs. LeDeuff and Renault [24], however, attributed some of the histopathology in internal organs, particularly kidneys, to the serious imbalance in osmoregulation resulting from skin lesions and fin lesions. In brief, it seems that LCD’s presentation in the gilthead seabream is genotype independent.

## 5. Conclusions

The sum of the data presented herein provides initial evidence that the outbreak involving one and a half million imported juvenile seabream was caused by LCDV genotype I. Despite the very low mortality rate that LCD causes, losses are enormous due to the unsightly fish appearance that would be difficult to commercialize, widespread skin lesions that can lead to imbalance in osmoregulation, potential alteration in swimming behavior due to fin lesions, and the lost energy spent in disease resistance and healing. Unfortunately, a broodstock can never be developed from the recovered fish since they become asymptomatic carriers of LCDV, and eggs spawned from these fish will be LCDV-positive as well as larvae hatching from them, as demonstrated by Cano and her colleagues [38]. Therefore, risk analysis is highly recommended for countries receiving live fish shipments to weigh the pros and cons of the introduction [9].

## Figures and Tables

**Table 1:** Reference sequences used in phylogenetic analysis.

GenBank Acc	Abbreviation	Genotype	Fish Species	Year	Origin
**MN128712**	LCDV-SA_EG	G-I	Gilthead seabream *(Sparus aurata)*	2017	(This study)
**L63545.1**	LCDV-1		European flounder *(Platichthys flesus)* Plaice *(Pleuronectes platessa L)*	1997	Europe
**GU939626.2**	YP-1	G-IX	Yellow perch *(Perca flavescens)*	2009	Canada
**GU290550.1**	Leetown NFH	G-VIII	Largemouth bass *(Micropterus salmoides)*	1962	USA
**AB299164.1**	PG06	G-VI	Pearl gourami *(Trichopodus leerii)*	2008	Korea
**AY849392.1**	KRF	G-III	Korean rock fish *(Sebastes schlegeli)*	2004	Korea
**AB213004.1**	RF03Yosu		*S. schlegeli*	2003	Korea
**KT438164.1**	LCDV-ss		*S. schlegeli*	2012	China
**AY823414.1**	RF		*S. schlegeli*	2004	Korea
**AB213000.1**	JF03Shinji	G-II	Japanese flounder *(P. olivaceus)*	2003	Korea
**AF126405.1**	Cn16		*P. olivaceus*	1999	China
**AY303804.1**	LCDV-K1		*P. olivaceus*	2003	Korea
**AY297741.1**	KLDV-1		*P. olivaceus*	2003	Korea
**AB212997.1**	JF00Kuma		*P. olivaceus*	2000	Japan
**AB212999.1**	JF00Yosu		*P. olivaceus*	2000	Korea
**AY380826.1**	LCDV-C		*P. olivaceus*	2003	China
**AY849391.1**	JF		*P. olivaceus*	2004	Korea
**KP184512.1**	PO6		*P. olivaceus*	2012	Korea
**AB212998.1**	JF03Yoshi		*P. olivaceus*	2003	Japan
**AB247938.1**	SB98Yosu	G-IV	Seabass *(Lateolabrax japonicus)*	2006	Korea
**EF059992.1**	RC		Cobia *(Rachycentron canadum)*	2006	China
**EF103188.1**	LCDV-RC		*R. canadum*	2006	China
**EF378607.1**	RC-Taiwan		*R. canadum*	2007	Taiwan
**AB299163.1**	PGF05	G-V	Painted glass fish *(Parambassis ranga)*	2007	Japan
**KJ408271.1**	LCDV-PF		Paradise fish *(Macropodus opercularis)*	2014	China
**GU320724.1**	SA3	G-VII	*S. aurata*	1997	Spain
**GU320731.1**	SA13		*S. aurata*	2005	Tunisia
**GU320734.1**	SA18		*S. aurata*	2008	Portugal
**EF184306.1**	SA-Eilat		*S. aurata*	2006	Israel
**GU320735.1**	SA19		*S. aurata*	2008	Spain
**GU320729.1**	SSE11		Senegalese sole *(Solea senegalensis)*	2001	Spain
**GU320730.1**	SA12		*S. aurata*	2003	Spain
**GU320726.1**	SA6		*S. aurata*	1998	Spain
**GU320727.1**	SA8		*S. aurata*	2000	Spain
**GU320725.1**	SA5		*S. aurata*	1998	Spain
**KX643370.1**	SA9		*S. aurata*	2001	Spain
**GU320736.1**	SSE20		*S. senegalensis*	2008	Spain
**GU320732.1**	SA14		*S. aurata*	2008	Spain
**HE650105.1**	SA1-ETun11		*S. aurata*	2011	Tunisia
**GU320739.1**	SA24		*S. aurata*	2009	France
**GU320737.1**	SA22		*S. aurata*	2009	France
**GU320738.1**	SA23		*S. aurata*	2009	France

**Table 2:** Amino acid identity and divergence between LCDV-SA_EG MCP sequence and representative sequences from other LCDV genotypes.

		Percent Identity									
		LCDV-SA_EG	(LCDV-1) G-I	(YP1) G-IX	(Leetown NFH) G-VIII	(PG06) G-VI	(RF) G-III	(JF03Yoshi) G-II	(LCDV-RC) G-IV	(SA-Eilat) G-VII	(PGF05) G-V
Divergence	LCDV-SA_EG		100	89.6	90.6	89.6	88.5	86.5	89.6	88.5	88.5
	(LCDV-1) G-I	0.0		89.6	90.6	89.6	88.5	86.5	89.6	88.5	88.5
	(YP1) G-IX	11.2	11.2		91.7	91.7	91.7	89.6	91.7	90.6	91.7
	(Leetown NFH) G-VIII	10.0	10.0	8.9		93.8	93.8	93.8	93.8	92.7	92.7
	(PG06) G-VI	11.2	11.2	8.9	6.5		93.8	92.7	91.7	91.7	90.6
	(RF) G-III	12.5	12.5	8.9	6.5	6.5		94.8	95.8	95.8	94.8
	(JF03Yoshi) G-II	15.0	15.0	11.2	6.5	7.7	5.4		95.8	95.8	94.8
	(LCDV-RC) G-IV	11.2	11.2	8.9	6.5	8.9	4.3	4.3		97.9	97.9
	(SA-Eilat) G-VII	12.5	12.5	10.0	7.7	8.9	4.3	4.3	2.1		99.0
	(PGF05) G-V	12.5	12.5	8.9	7.7	10.0	5.4	5.4	2.1	1.0	

## Supplementary Materials

The following are available online at https://www.mdpi.com/article/10.3390/ani11113032/s1, Figure S1: PCR results of partial MCP gene amplification from LCDV in field samples, Figure S2: SnapGene ®-generated multiple alignment showing consensus sequence and sequence mismatches, Figure S3: BLASTn alignment results showing graphic summary and alignment as flat query-anchored with dots for identities, Table S1: Tabulated BLASTn alignment results.
