# Supplementary material for: Initial Evidence That Gilthead Seabream (Sparus aurata L.) Is a Host for Lymphocystis Disease Virus Genotype I
Source: Animals (Basel). 2021 Oct 22;11(11):3032. doi: 10.3390/ani11113032 (PMC8614504; doi:10.3390/ani11113032)
Supplement: Supplementary file 1 [file animals-11-03032-s001.zip › animals-1331593-supplementary Figure S1 PCR results LCDV in field samples..pdf]

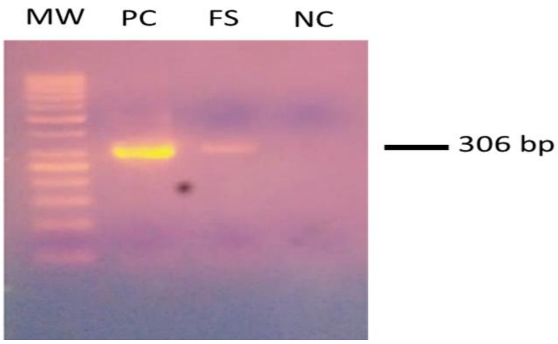

Figure S1. PCR result of partial amplification of the MCP gene of LCDV in field samples. DNA extracted from pooled nodules on an infected fish was tested for the presence of LCDV DNA using the developed assay. MW: GeneRuler® 50 bp DNA Ladder (Thermo Scientific™). PC: Synthetic positive control ($9X10^{-7}$ genome copy equivalents/ul). FS: Field sample. NC: DH5α bacterial DNA extract (negative control).
