# Supplementary material for: Initial Evidence That Gilthead Seabream (Sparus aurata L.) Is a Host for Lymphocystis Disease Virus Genotype I
Source: Animals (Basel). 2021 Oct 22;11(11):3032. doi: 10.3390/ani11113032 (PMC8614504; doi:10.3390/ani11113032)
Supplement: Supplementary file 1 [file animals-11-03032-s001.zip › animals-1331593-supplementary Figure S2 SnapGene generated multiple alignment showing consensus sequence and sequence mismatches.pdf]

Aligned using an external algorithm

Consensus

1. LCDV-SA_EG
2. LCDV-1
3. PG06
4. LeetownNFH
5. YP1
6. JF03Yoshi
7. PO6
8. JF
9. LCDV-C RC
10. JF00Yosu
11. JF00Kuma
12. KLDV-1
13. LCDV-K1
14. Cn16
15. JF03Shinji
16. RF
17. LCDV-ss RC
18. RF03Yosu
19. KRF
20. LCDV-RC
21. SB98Yosu
22. RC
23. RC-Taiwan
24. LCDV-PF
25. PGF05
26. SA12
27. SA18
28. SA13
29. SA3
30. SA.Eilat
31. SA1.ETun11
32. SA24
33. SA23
34. SA22
35. SA19
36. SA9
37. SSE20
38. SA14
39. SA5
40. SA8
41. SA6
42. SSE11

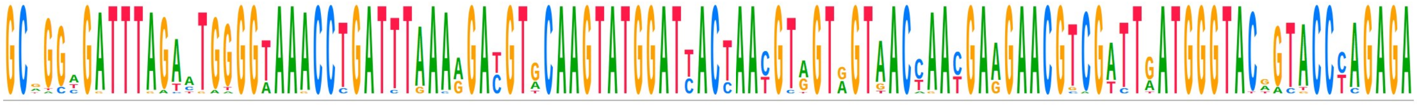

	GCNGGNGATTTAGAATGGGGTAAACCTGATTTAAAAGATGTGCAAGTATGGATTACTAACGTAGTNGTAACCAACGAAGAACGTCGATTGATGGGTACNGTACCCAGAGA	
1	GCTGCTGATTTGGAATATGGAAAACCTGATTTAAAAGACGTTCAAGTATGGATTACTAATGCAGTGGTTACAAATGAAGAACGGCGACTTATGGGTACAACTCCTCGAGA	110
2	GCTGCTGATTTAGAATATGGAAAACCTGATTTAAAAGACGTTCAAGTATGGATTACTAATGCAGTGGTTACAAATGAAGAACGGCGACTTATGGGTACAACTCCTCGAGA	110
3	GCTAGTGATTTAGTAGGAGGAAAACCTGATTTAAAAGATGTACAAGTATGGATTACTAACGTAGTAGTAACCAATGAAGAACGTAGATTAATGGGTACCGTACCTCGAGA	110
4	GCCGCTGATTTAATGTGGGGAAAACCCGATCTAAAAGATGTTCAAGTATGGATTACTAACGTAGTTGTAACCAACGAAGAACGCCGACTAATGGGTACTGTACCTAGAGA	110
5	GCGTCTGATTTAACTTGGGGAAAACCCGATCTAACCGACGTTCAAGTATGGATTACTAACGTAGTCGTAACTAACGAAGAACGTCGATTGATGGGTATTACGCCTAGAGA	110
6	GCAGGCGATTTAGACTGGGGTAAACCTGATTTAAAGGATGTGCAAGTATGGATTACTAATGTAGTAGTAACCAATGAGGAACGTCGTTTAATGGGTACAGTACCTAGAGA	110
7	GCAGGCGATTTAGACTGGGGTAAACCTGATTTAAAGGATGTGCAAGTATGGATTACTAATGTAGTAGTAACCAATGAGGAACGTCGTTTAATGGGTACAGTACCTAGAGA	110
8	GCAGGCGATTTAGACTGGGGTAAACCTGATTTAAAGGATGTGCAAGTATGGATTACTAATGTAGTAGTAACCAATGAGGAACGTCGTTTAATGGGTACAGTACCTAGAGA	110
9	GCAGGCGATTTAGACTGGGGTAAACCTGATTTAAAGGATGTGCAAGTATGGATTACTAATGTAGTAGTAACCAATGAGGAACGTCGTTTAATGGGTACAGTACCTAGAGA	110
10	GCAGGCGATTTAGACTGGGGTAAACCTGATTTAAAGGATGTGCAAGTATGGATTACTAATGTAGTAGTAACCAATGAGGAACGTCGTTTAATGGGTACAGTACCTAGAGA	110
11	GCAGGCGATTTAGACTGGGGTAAACCTGATTTAAAGGATGTGCAAGTATGGATTACTAATGTAGTAGTAACCAATGAGGAACGTCGTTTAATGGGTACAGTACCTAGAGA	110
12	GCAGGCGATTTAGACTGGGGTAAACCTGATTTAAAGGATGTGCAAGTATGGATTACTAATGTAGTAGTAACCAATGAGGAACGTCGTTTAATGGGTACAGTACCTAGAGA	110
13	GCAGGCGATTTAGACTGGGGTAAACCTGATTTAAAGGATGTGCAAGTATGGATTACTAATGTAGTAGTAACCAATGAGGAACGTCGTTTAATGGGTACAGTACCTAGAGA	110
14	GCAGGCGATTTAGACTGGGGTAAACCTGATTTAAAGGATGTGCAAGTATGGATTACTAATGTAGTAGTAACCAATGAGGAACGTCGTTTAATGGGTACAGTACCTAGAGA	110
15	GCAGGCGATTTAGACTGGGGTAAGCCTGATTTAAAGGATGTGCAAGTATGGATTACTAATGTAGTAGTAACCAATGAGGAACGTCGTTTAATGGGTACAGTACCTAGAGA	110
16	GCTGGAGGTTTAGTTTGGGGAAAACCTGATTTAAAAGATGTACAAGTATGGATTACCAATGTAGTAGTAACTAATGAAGAACGTCGATTGATGGGTACTGTACCTAGAGA	110
17	GCTGGAGATTTAGTTTGGGGAAAACCTGATTTAAAAGATGTACAAGTATGGATTACCAATGTAGTAGTAACTAATGAAGAACGTCGATTGATGGGTACTGTACCTAGAGA	110
18	GCTGGAGATTTAGTTTGGGGAAAACCTGATTTAAAAGATGTACAAGTATGGATTACCAATGTAGTAGTAACTAATGAAGAACGTCGATTGATGGGTACTGTACCTAGAGA	110
19	GCTGGAGATTTAGTTTGGGGAAAACCTGATTTAAAAGATGTACAAGTATGGATTACCAATGTAGTAGTAACTGATGAAGAACGTCGATTGATGGGTACTGTACCTAGAGA	110
20	GCAGGTGATTTAGAATGGGGTAAACCTGATTTGAAGGATGTACAAGTATGGATTACCAACGTAGTAGTAACTAACGAAGAACGTCGATTGATGGGTACAGTACCCAGAGA	110
21	GCAGGTGATTTAGAATGGGGTAAACCTGATTTGAAGGATGTACAAGTATGGATTACCAACGTAGTAGTAACTAACGAAGAACGTCGATTGATGGGTACAGTACCCAGAGA	110
22	GCAGGTGATTTAGAATGGGGTAAACCTGATTTGAAGGATGTACAAGTATGGATTACCAACGTAGTAGTAACTAACGAAGAACGTCGATTGATGGGTACAGTACCCAGAGA	110
23	GCAGGTGATTTAGAATGGGGTAAACCTGATTTGAAGGATGTACAAGTATGGATTACCAACGTAGTAGTAACTAACGAAGAACGTCGATTGATGGGTACAGTACCCAGAGA	110
24	GCGGGTGATTTAGAATGGGGTAAACCCGATTTAAAAGATGTACAAGTATGGATCACTAACGTTGTGGTGACCAACGAAGAACGTCGATTGATGGGTACGATACCTAGAGA	110
25	GCCGGTGATTTAGAGTGGGGTAAACCTGATTTAAAGGATGTACAAGTATGGATCACCAATGTTGTAGTGACTAATGAAGAACGTCGATTGATGGGTACGGTACCCAGAGA	110
26	GCGGGAGATTTAGAATGGGGTAAACCTGATTTAAAAGACGTGCAAGTATGGATCACTAACGTGGTGGTAACCAACGAAGAACGTCGATTGATGGGTACGGTACCCAGAGA	110
27	GCGGGAGATTTAGAATGGGGTAAACCTGATTTAAAAGACGTGCAAGTATGGATTACTAACGTGGTGGTGACCAACGAAGAACGTCGATTGATGGGTACGGTACCCAGAGA	110
28	GCGGGAGATTTAGAATGGGGTAAACCTGATTTAAAAGACGTGCAAGTATGGATTACTAACGTGGTGGTGACCAACGAAGAACGTCGATTGATGGGTACGGTACCCAGAGA	110
29	GCGGGAGATTTAGAATGGGGTAAACCTGATTTAAAAGACGTGCAAGTATGGATTACTAACGTGGTGGTGACCAACGAAGAACGTCGATTGATGGGTACGGTACCCAGAGA	110
30	GCGGGAGATTTAGAATGGGGTAAACCTGATTTAAAAGACGTGCAAGTATGGATCACTAACGTAGTGGTAACCAACGAAGAACGTCGATTGATGGGTACGGTACCCAGAGA	110
31	GCGGGAGATTTAGAATGGGGTAAACCTGATTTAAAAGACGTGCAAGTATGGATCACTAACGTGGTGGTAACTAACGAAGAACGTCGATTGATGGGTACGGTACCCAGAGA	110
32	GCGGGAGATTTAGAATGGGGTAAACCTGATTTAAAAGACGTGCAAGTATGGATCACTAACGTGGTGGTAACTAACGAAGAACGTCGATTGATGGGTACGGTACCCAGAGA	110
33	GCGGGAGATTTAGAATGGGGTAAACCTGATTTAAAAGACGTGCAAGTATGGATCACTAACGTGGTGGTAACTAACGAAGAACGTCGATTGATGGGTACGGTACCCAGAGA	110
34	GCGGGAGATTTAGAATGGGGTAAACCTGATTTAAAAGACGTGCAAGTATGGATCACTAACGTGGTGGTAACTAACGAAGAACGTCGATTGATGGGTACGGTACCCAGAGA	110
35	GCGGGAGATTTAGAATGGGGTAAACCTGATTTAAAAGACGTGCAAGTATGGATTACTAACGTGGTGGTAACCAACGAAGAACGTCGATTGATGGGTACGGTACCCAGAGA	110
36	GCGGGAGATTTAGAATGGGGTAAACCTGATTTAAAAGACGTGCAAGTATGGATCACTAACGTGGTGGTAACCAACGAAGAACGTCGATTGATGGGTACGGTACCCAGAGA	110
37	GCGGGAGATTTAGAATGGGGTAAACCTGATTTAAAAGACGTGCAAGTATGGATCACTAACGTGGTGGTAACCAACGAAGAACGTCGATTGATGGGTACGGTACCCAGAGA	110
38	GCGGGAGATTTAGAATGGGGTAAACCTGATTTAAAAGACGTGCAAGTATGGATCACTAACGTGGTGGTAACCAACGAAGAACGTCGATTGATGGGTACGGTACCCAGAGA	110
39	GCGGGAGATTTAGAATGGGGTAAACCTGATTTAAAAGACGTGCAAGTATGGATCACTAACGTGGTGGTAACCAACGAAGAACGTCGATTGATGGGTACGGTACCCAGAGA	110
40	GCGGGAGATTTAGAATGGGGTAAACCTGATTTAAAAGACGTGCAAGTATGGATCACTAACGTGGTGGTAACCAACGAAGAACGTCGATTGATGGGTACGGTACCCAGAGA	110
41	GCGGGAGATTTAGAATGGGGTAAACCTGATTTAAAAGACGTGCAAGTATGGATCACTAACGTGGTGGTAACCAACGAAGAACGTCGATTGATGGGTACGGTACCCAGAGA	110
42	GCGGGAGATTTAGAATGGGGTAAACCTGATTTAAAAGACGTGCAAGTATGGATCACTAACGTGGTGGTAACCAACGAAGAACGTCGATTGATGGGTACGGTACCCAGAGA	110

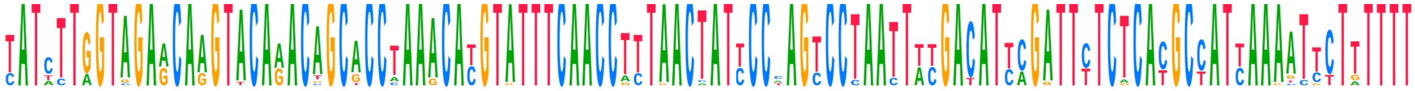

TATCTTGGTAGAACAAGTACAAACAGCACCTAAACATGTATTTCAACCTTTAACTATTCCNAGTCCTAATTTTGACATTCGATTCTCTCACGCCATTAAAATTCTTTTTT

120 130 140 150 160 170 180 190 200 210 220

1	TATATTAGTGGAACAAGTTCAAACTGCGCCAAAACATGTATTTCAACCGTTAACTATTCCAAGCCCTAATTTTGATATTCGGTTTTCACACGCTATTAAATTGTTATTTT	220
2	TATATTAGTGGAACAAGTTCAAACTGCGCCAAAACATGTATTTCAACCGTTAACTATTCCAAGCCCTAATTTTGATATTCGGTTTTCACACGCTATTAAATTGTTATTTT	220
3	TATATTAGTAGAACAAGTACAAACAGCGCCGAAACATGTATTTCAACCTTTAACTATTCCTAGTCCTAATTTTGATATTAGATTTTCTCACGCTATTAAAATTTTATTTT	220
4	TATTTTGGTAGAACAAGTACAAACTGCTCCAAAACACGTATTTCAACCTTTAACAATCCCGAGTCCTAACTTTGATATTCGATTCTCTCACGCTATTAAGCTTTTATTTT	220
5	TATTTTAGTCGAACAAGTACAAACCGCTCCAAAACACGTATTTCAACCTTTAACTATTCCTAGCCCTAATTTCGATATCCGATTTTCGCACGCTATTAAAACTCTGTTTT	220
6	CATCTTGGTAGAACAGGTACAAACAGCACCTAAACATGTATTTCAACCTCTAACTATTCCAAGTCCTAATTTTGACATCAGATTTTCTCATGCCATTAAAATCCTTTTTT	220
7	CATCTTGGTAGAACAGGTACAAACAGCACCTAAACATGTATTTCAACCTCTAACTATTCCAAGTCCTAATTTTGACATCAGATTTTCTCATGCCATTAAAATCCTTTTTT	220
8	CATCTTGGTAGAACAGGTACAAACAGCACCTAAACATGTATTTCAACCTCTAACTATTCCAAGTCCTAATTTTGACATCAGATTTTCTCATGCCATTAAAATCCTTTTTT	220
9	CATCTTGGTAGAACAGGTACAAACAGCACCTAAACATGTATTTCAACCTCTAACTATTCCAAGTCCTAATTTTGACATCAGATTTTCTCATGCCATTAAAATCCTTTTTT	220
10	CATCTTGGTAGAACAGGTACAAACAGCACCTAAACATGTATTTCAACCTCTAACTATTCCAAGTCCTAATTTTGACATCAGATTTTCTCATGCCATTAAAATCCTTTTTT	220
11	CATCTTGGTAGAACAGGTACAAACAGCACCTAAACATGTATTTCAACCTCTAACTATTCCAAGTCCTAATTTTGACATCAGATTTTCTCATGCCATTAAAATCCTTTTTT	220
12	CATCTTGGTAGAACAGGTACAAACAGCACCTAAACATGTATTTCAACCTCTAACTATTCCAAGTCCTAATTTTGACATCAGATTTTCTCATGCCATTAAAATCCTTTTTT	220
13	CATCTTGGTAGAACAGGTACAAACAGCACCTAAACATGTATTTCAACCTCTAACTATTCCAAGTCCTAATTTTGACATCAGATTTTCTCATGCCATTAAAATCCTTTTTT	220
14	CATCTTGGTAGAACAGGTACAAACAGCACCTAAACATGTATTTCAACCTCTAACTATTCCAAGTCCTAATTTTGACATCAGATTTTCTCATGCCATTAAAATCCTTTTTT	220
15	CATCTTGGTAGAACAGGTACAAACAGCACCTAAACATGTATTTCAACCTCTAACTATTCCAAGTCCTAATTTTGACATCAGATTTTCTCATGCCATTAAAATCCTTTTTT	220
16	TATACTAGTAGAACAAGTACAAACAGCACCTAAACATGTATTTCAACCATTAACTATTCCTAGCCCTAACTTTGACATTCGATTCTCTCATGCTATTAAAATTTTGTTTT	220
17	TATACTAGTAGAACAAGTACAAACAGCACCTAAACATGTATTTCAACCATTAACTATTCCTAGCCCTAACTTTGACATTCGATTCTCTCATGCTATTAAAATTTTGTTTT	220
18	TATACTAGTAGAACAAGTACAAACAGCACCTAAACATGTATTTCAACCATTAACTATTCCTAGCCCTAACTTTGACATTCGATTCTCTCATGCTATTAAAATTTTGTTTT	220
19	TATACTAGTAGAACAAGTACAAACAGCACCTAAACATGTATTTCAACCATTAACTATTCCTAGCCCTAACTTTGACATTCGATTCTCTCATGCTATTAAAATTTTGTTTT	220
20	TATTTTGGTAGAACAAGTACAAACAGCGCCCAAGCATGTATTTCAACCTTTAACTATTCCTAGCCCTAATTTTGACATCCGATTCTCTCACGCTATTAAAGTTCTTTTTT	220
21	TATTTTGGTAGAACAAGTACAAACAGCGCCTAAGCATGTATTTCAACCTTTAACTATTCCTAGCCCTAATTTTGACATCCGATTTTCTCACGCTATTAAAATCCTTTTTT	220
22	TATTTTGGTAGAACAAGTACAAACAGCGCCCAAGCATGTATTTCAACCTTTAACTATTCCTAGCCCTAATTTTGACATCCGATTCTCTCACGCTATTAAAGTTCTTTTTT	220
23	TATTTTGGTAGAACAAGTACAAACAGCGCCCAAGCATGTATTTCAACCTTTAACTATTCCTAGCCCTAATTTTGACATCCGATTCTCTCACGCTATTAAAGTTCTTTTTT	220
24	TATTTTAGTAGAGCAAGTACAAACGGCGCCTAAACACGTATTTCAACCTTTAACCATTCCAAGTCCCAATTACGACATCAGATTTTCTCACGCCATCAAAACTCTTTTTT	220
25	TATTTTGGTAGAACAAGTACAAACAGCACCAAAACATGTGTTTCAACCATTGACTATTCCTAGTCCTAATTATGACATTAGATTTTCTCATGCCATTAAAACTCTTTTTT	220
26	TATCTTGGTAGAGCAAGTACAGACAGCACCTAAACACGTATTTCAACCTTTAACTATCCCCAGTCCCAATTACGACATTCGATTCTCTCACGCCATCAAAATTCTTTTTT	220
27	TATCTTGGTAGAGCAAGTACAGACAGCACCTAAACACGTATTTCAACCTTTAACTATCCCCAGTCCTAATTACGACATTCGATTCTCTCACGCCATCAAAATTCTTTTTT	220
28	TATCTTGGTAGAGCAAGTACAGACAGCACCTAAACACGTATTTCAACCTTTAACTATCCCCAGTCCTAATTACGACATTCGATTCTCTCACGCCATCAAAATTCTTTTTT	220
29	TATCTTGGTAGAGCAAGTACAGACAGCACCTAAACACGTATTTCAACCTTTAACTATCCCCAGTCCTAATTACGACATTCGATTCTCTCACGCCATCAAAATTCTTTTTT	220
30	TATCTTGGTAGAGCAAGTACAGACAGCACCTAAACACGTATTTCAACCTTTAACTATCCCCAGTCCTAATTACGACATTCGATTCTCTCACGCCATCAAAATTCTTTTTT	220
31	TATCTTGGTAGAGCAAGTACAGACAGCACCTAAACACGTATTTCAACCTTTAACTATCCCCAGTCCTAATTACGACATTCGATTCTCTCACGCCATCAAAATTCTTTTTT	220
32	TATCTTGGTAGAGCAAGTACAGACAGCACCTAAACACGTATTTCAACCTTTAACTATCCCCAGTCCTAATTACGACATTCGATTCTCTCACGCCATCAAAATTCTTTTTT	220
33	TATCTTGGTAGAGCAAGTACAGACAGCACCTAAACACGTATTTCAACCTTTAACTATCCCCAGTCCTAATTACGACATTCGATTCTCTCACGCCATCAAAATTCTTTTTT	220
34	TATCTTGGTAGAGCAAGTACAGACAGCACCTAAACACGTATTTCAACCTTTAACTATCCCCAGTCCTAATTACGACATTCGATTCTCTCACGCCATCAAAATTCTTTTTT	220
35	TATCTTGGTAGAGCAAGTACAGACAGCACCTAAACACGTATTTCAACCTTTAACTATCCCCAGTCCTAATTACGACATTCGATTCTCTCACGCCATCAAAATTCTTTTTT	220
36	TATCTTGGTAGAGCAAGTACAGACAGCACCTAAACACGTATTTCAACCTTTAACTATCCCCAGTCCTAATTACGACATTCGATTCTCTCACGCCATCAAAATTCTTTTTT	220
37	TATCTTGGTAGAGCAAGTACAGACAGCACCTAAACACGTATTTCAACCTTTAACTATCCCCAGTCCTAATTACGACATTCGATTCTCTCACGCCATCAAAATTCTTTTTT	220
38	TATCTTGGTAGAGCAAGTACAGACAGCACCTAAACACGTATTTCAACCTTTAACTATCCCCAGTCCTAATTACGACATTCGATTCTCTCACGCCATCAAAATTCTTTTTT	220
39	TATCTTGGTAGAGCAAGTACAGACAGCACCTAAACACGTATTTCAACCTTTAACTATCCCCAGTCCTAATTACGACATTCGATTCTCTCACGCCATCAAAATTCTTTTTT	220
40	TATCTTGGTAGAGCAAGTACAGACAGCACCTAAACACGTATTTCAACCTTTAACTATCCCCAGTCCTAATTACGACATTCGATTCTCTCACGCCATCAAAATTCTTTTTT	220
41	TATCTTGGTAGAGCAAGTACAGACAGCACCTAAACACGTATTTCAACCTTTAACTATCCCCAGTCCTAATTACGACATTCGATTCTCTCACGCCATCAAAATTCTTTTTT	220
42	TATCTTGGTAGAGCAAGTACAGACAGCACCTAAACACGTATTTCAACCTTTAACTATCCCCAGTCCCAATTACGACATTCGATTCTCTCACGCCATCAAAATTCTTTTTT	220

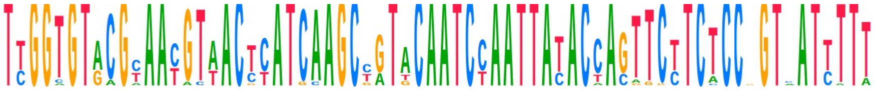

TTGGTGTACGNAACGTAACTCATCAAGCNGTACAATCCAATTATACCAGTTCTTCTCCNGTNATTTTT

230 240 250 260 270 280

1	TTGGTGTAAGAAATACAACTCATGCAGCTGTTCAATCTAATTATACTACAGCTTCTCCTGTTATTTTA	288
2	TTGGTGTAAGAAATACAACTCATGCAGCTGTTCAATCTAATTATACTACAGCTTCTCCTGTTATTTTA	288
3	TTGGCGTACGCAACGTAACTTATCAAGCCGTGCAATCTAATTACACTACGTGTTCACCCGTAATTTTA	288
4	TTGGAGTACGAAATGTAACGTATCAAGCGGTACAATCTAATTATACAACTTCTTCTCCCGTTATTATA	288
5	TTGGTGTAAGAAACGTAACGCATCAAGCGGTACAATCTAATTATACAACTTCTTCTCCCGTTATTTTA	288
6	TCGGTGTGCGTAATGTTACCTATCAAGCTATACAATCCAATTACACCAGTTCTTCTCCTGTAATTTTT	288
7	TCGGTGTGCGTAATGTTACCTATCAAGCTATACAATCCAATTACACCAGTTCTTCTCCTGTAATCTTT	288
8	TCGGTGTGCGTAATGTTACCTATCAAGCTATACAATCCAATTACACCAGTTCTTCTCCTGTAATCTTT	288
9	TCGGTGTGCGTAATGTTACCTATCAAGCTATACAATCCAATTACACCAGTTCTTCTCCTGTAATCTTT	288
10	TCGGTGTGCGTAATGTTACCTATCAAGCTATACAATCCAATTACACCAGTTCTTCTCCTGTAATCTTT	288
11	TCGGTGTGCGTAATGTTACCTATCAAGCTATACAATCCAATTACACCAGTTCTTCTCCTGTAATCTTT	288
12	TCGGTGTGCGTAATGTTACCTATCAAGCTATACAATCCAATTACACCAGTTCTTCTCCTGTAATCTTT	288
13	TCGGTGTGCGTAATGTTACCTATCAAGCTATACAATCCAATTACACCAGTTCTTCTCCTGTAATCTTT	288
14	TCGGTGTGCGTAATGTTACCTATCAAGCTATACAATCCAATTACACCAGTTCTTCTCCTGTAATCTTT	288
15	TCGGTGTGCGTAATGTTACCTATCAAGCTATACAATCCAATTACACCAGTTCTTCTCCTGTAATCTTT	288
16	TCGGTGTGCGTAATGTAACTCATCAAGCTGTTCAATCTAATTATACTACTTCTTCACCAGTGATTTTT	288
17	TTGGTGTGCGTAATGTAACTCATCAAGCTGTTCAATCTAATTATACTACTTCTTCACCAGTGATTTTT	288
18	TTGGTGTGCGTAATGTAACTCATCAAGCTGTTCAATCTAATTATACTACTTCTTCACCAGTGATTTTT	288
19	TTGGTGTGCGTAATGTAACTCATCAAGCTGTTCAATCTAATTATACTACTTCTTCACCAGTGATTTTT	288
20	TTGGTGTACGTAACGTTACTCATCAAGCGGTACAATCCAATTATACCAGTTCTTCTCCCGTAATCTTT	288
21	TCGGTGTACGCAACGTTACTCATCAAGCGGTACAATCCAATTATACCAGTTCTTCTCCTGTAATCTTT	288
22	TTGGTGTACGTAACGTTACTCATCAAGCGGTACAATCCAATTATACCAGTTCTTCTCCCGTAATCTTT	288
23	TTGGTGTACGTAACGTTACTCATCAAGCGGTACAATCCAATTATACCAGTTCTTCTCCCGTAATCTTT	288
24	TCGGCGTACGCAACGTAACTCATCAAGCCGTACAATCCAATTATACCAGTTCTTCCCCGGTTATTTTT	288
25	TCGGTGTACGCAATGTAACTCATCAAGCTGTACAATCCAATTATACCAGTTCTTCTCCGGTCATTTTT	288
26	TTGGTGTACGCAACGTAACTCATCAAGCCGTACAATCCAATTATACCAGTTCCTCTCCGGTCATTTTT	288
27	TTGGTGTACGCAACGTAACTCATCAAGCCGTACAATCCAATTATACCAGTTCTTCTCCGGTCATTTTT	288
28	TTGGTGTACGCAACGTAACTCATCAAGCCGTACAATCCAATTATACCAGTTCTTCTCCGGTCATTTTT	288
29	TTGGTGTACGCAACGTAACTCATCAAGCCGTACAATCCAATTATACCAGTTCTTCTCCGGTCATTTTT	288
30	TTGGTGTACGCAACGTAACTCATCAAGCCGTACAATCCAATTATACCAGTTCTTCTCCGGTCATTTTT	288
31	TTGGTGTACGCAACGTAACTCATCAAGCCGTGCAATCTAATTATACCAGTTCCTCTCCGGTCATTTTT	288
32	TTGGTGTACGCAACGTAACTCATCAAGCCGTGCAATCTAATTATACCAGTTCCTCTCCGGTCATTTTT	288
33	TTGGTGTACGCAACGTAACTCATCAAGCCGTGCAATCTAATTATACCAGTTCCTCTCCGGTCATTTTT	288
34	TTGGTGTACGCAACGTAACTCATCAAGCCGTGCAATCTAATTATACCAGTTCCTCTCCGGTCATTTTT	288
35	TTGGTGTACGCAACGTAACTCATCAAGCCGTACAATCCAATTATACCAGTTCCTCTCCGGTCATTTTT	288
36	TTGGTGTACGCAACGTAACTCATCAAGCCGTACAATCCAATTATACCAGTTCCTCTCCGGTCATTTTT	288
37	TTGGTGTACGCAACGTAACTCATCAAGCCGTACAATCCAATTATACCAGTTCCTCTCCGGTCATTTTT	288
38	TTGGTGTACGCAACGTAACTCATCAAGCCGTACAATCCAATTATACCAGTTCCTCTCCGGTCATTTTT	288
39	TTGGTGTACGCAACGTAACTCATCAAGCCGTACAATCCAATTATACCAGTTCCTCTCCGGTCATTTTT	288
40	TTGGTGTACGCAACGTAACTCATCAAGCCGTACAATCCAATTATACCAGTTCCTCTCCGGTCATTTTT	288
41	TTGGTGTACGCAACGTAACTCATCAAGCCGTACAATCCAATTATACCAGTTCCTCTCCGGTCATTTTT	288
42	TTGGTGTACGCAACGTAACTCATCAAGCCGTACAATCCAATTATACCAGTTCCTCTCCGGTCATTTTT	288

Sequence Logo: 50% GC base composition

Consensus Threshold: >50%

Compare to: LCDV-SA_EG

Bases that match the reference are marked with gray highlighting.

Created: Sep 15, 2021

Last Modified: Sep 15, 2021
