# Supplementary material for: Initial Evidence That Gilthead Seabream (Sparus aurata L.) Is a Host for Lymphocystis Disease Virus Genotype I
Source: Animals (Basel). 2021 Oct 22;11(11):3032. doi: 10.3390/ani11113032 (PMC8614504; doi:10.3390/ani11113032)
Supplement: Supplementary file 1 [file animals-11-03032-s001.zip › animals-1331593-Supplementary Figure S3 BLASTn alignment results showing graphic summary and alignment as flat query-anchored with dots for identities W.pdf]

RID: TR0S41W9016
Job Title:Nucleotide Sequence
Program: BLASTN
Query: None ID: lcl|Query_6709(dna) Length: 288
Database: nt Nucleotide collection (nt)

Sequences producing significant alignments:

| Description | Scientific Name | Common Name | Taxid | Max Score | Total Score | Query cover | E Value | Per. Ident | Acc. Len | Accession |
|---|---|---|---|---|---|---|---|---|---|---|
| Lymphocystis disease virus 1 major capsid protein gene, partia... | Lymphocystis... | NA | 36363 | 520 | 520 | 100% | 3e-143 | 100.00 | 288 | MN128712.1 |
| Lymphocystis disease virus 1, complete genome | Lymphocystis... | NA | 36363 | 516 | 516 | 100% | 1e-141 | 99.65 | 102653 | L63545.1 |
| Lymphocystis disease virus 1 strain LCDV-ss major capsid prote... | Lymphocystis... | NA | 36363 | 288 | 288 | 99% | 3e-73 | 82.23 | 848 | KT438164.1 |
| Lymphocystis disease virus 1 MCP gene for major capsid protein... | Lymphocystis... | NA | 36363 | 288 | 288 | 99% | 3e-73 | 82.23 | 1356 | AB213004.1 |
| Lymphocystis disease virus 1 MCP gene for major capsid protein... | Lymphocystis... | NA | 36363 | 286 | 286 | 98% | 1e-72 | 82.33 | 1337 | AB299164.1 |
| Lymphocystis disease virus from Sebastes schlegeli major capsi... | Lymphocystis... | NA | 36363 | 284 | 284 | 99% | 4e-72 | 81.88 | 1380 | AY849392.1 |
| Lymphocystis disease virus 1 major capsid protein (MCP) gene,... | Lymphocystis... | NA | 36363 | 283 | 283 | 93% | 1e-71 | 83.27 | 1380 | AY823414.1 |
| Lymphocystis disease virus 1 isolate yellow perch major capsid... | Lymphocystis... | NA | 36363 | 280 | 280 | 93% | 4e-71 | 82.96 | 1357 | GU939626.2 |
| Lymphocystis disease virus 1 strain Leetown NFH major capsid... | Lymphocystis... | NA | 36363 | 270 | 270 | 100% | 8e-68 | 81.03 | 1317 | GU290550.1 |
| Lymphocystis disease virus 1 MCP gene for major capsid protein... | Lymphocystis... | NA | 36363 | 248 | 248 | 97% | 3e-61 | 79.43 | 1356 | AB247938.1 |
| Lymphocystis disease virus 4 isolate LCDV-WC, complete genome | Lymphocystis... | NA | 2704413 | 244 | 244 | 97% | 3e-60 | 79.29 | 211086 | NC_055603.1 |
| Lymphocystis disease virus 1 major capsid protein gene, partia... | Lymphocystis... | NA | 36363 | 243 | 243 | 97% | 1e-59 | 79.08 | 1356 | EF059992.1 |
| Lymphocystis disease virus 1 major capsid protein gene, comple... | Lymphocystis... | NA | 36363 | 243 | 243 | 97% | 1e-59 | 79.08 | 1380 | EF378607.1 |
| Lymphocystis disease virus 1 strain LCDV-RC major capsid prote... | Lymphocystis... | NA | 36363 | 243 | 243 | 97% | 1e-59 | 79.08 | 1380 | EF103188.1 |
| Lymphocystis disease virus WC major capsid protein gene, parti... | Lymphocystis... | NA | 2603837 | 237 | 237 | 90% | 5e-58 | 80.08 | 1355 | MK250973.1 |
| Lymphocystis disease virus 1 MCP gene for major capsid protein... | Lymphocystis... | NA | 36363 | 230 | 230 | 97% | 7e-56 | 78.01 | 1337 | AB299163.1 |
| Lymphocystis disease virus 1 isolate SA18 major capsid protein... | Lymphocystis... | NA | 36363 | 228 | 228 | 97% | 2e-55 | 77.94 | 1317 | GU320734.1 |
| Lymphocystis disease virus 1 isolate SA13 major capsid protein... | Lymphocystis... | NA | 36363 | 228 | 228 | 97% | 2e-55 | 77.94 | 1215 | GU320731.1 |
| Lymphocystis disease virus 1 isolate SA3 major capsid protein... | Lymphocystis... | NA | 36363 | 228 | 228 | 97% | 2e-55 | 77.94 | 1278 | GU320724.1 |
| Lymphocystis disease virus 1 isolate LCDV-SA-Eilat major capsi... | Lymphocystis... | NA | 36363 | 228 | 228 | 97% | 2e-55 | 77.94 | 1337 | EF184306.1 |
| Lymphocystis disease virus 1 partial MCP gene for major capsid... | Lymphocystis... | NA | 36363 | 224 | 224 | 97% | 1e-53 | 77.58 | 1317 | HE650105.1 |
| Lymphocystis disease virus 1 isolate SA24 major capsid protein... | Lymphocystis... | NA | 36363 | 224 | 224 | 97% | 1e-53 | 77.58 | 1317 | GU320739.1 |
| Lymphocystis disease virus 1 isolate SA23 major capsid protein... | Lymphocystis... | NA | 36363 | 224 | 224 | 97% | 1e-53 | 77.58 | 1140 | GU320738.1 |
| Lymphocystis disease virus 1 isolate SA22 major capsid protein... | Lymphocystis... | NA | 36363 | 224 | 224 | 97% | 1e-53 | 77.58 | 1275 | GU320737.1 |
| Lymphocystis disease virus 1 isolate SA19 major capsid protein... | Lymphocystis... | NA | 36363 | 224 | 224 | 97% | 1e-53 | 77.58 | 1317 | GU320735.1 |
| Lymphocystis disease virus 1 isolate LCDV-PF major capsid... | Lymphocystis... | NA | 36363 | 221 | 221 | 97% | 4e-53 | 77.30 | 1208 | KJ408271.1 |
| Lymphocystis disease virus Sa isolate SA9, complete genome | Lymphocystis... | NA | 1898060 | 219 | 219 | 97% | 1e-52 | 77.22 | 208501 | KX643370.1 |
| Lymphocystis disease virus 1 isolate SSE20 major capsid protei... | Lymphocystis... | NA | 36363 | 219 | 219 | 97% | 1e-52 | 77.22 | 1317 | GU320736.1 |
| Lymphocystis disease virus 1 isolate SA14 major capsid protein... | Lymphocystis... | NA | 36363 | 219 | 219 | 97% | 1e-52 | 77.22 | 1317 | GU320732.1 |
| Lymphocystis disease virus 1 isolate SA5 major capsid protein... | Lymphocystis... | NA | 36363 | 219 | 219 | 97% | 1e-52 | 77.22 | 1317 | GU320725.1 |
| Lymphocystis disease virus 1 isolate SA8 major capsid protein... | Lymphocystis... | NA | 36363 | 219 | 219 | 97% | 1e-52 | 77.22 | 1296 | GU320727.1 |
| Lymphocystis disease virus 1 isolate SA6 major capsid protein... | Lymphocystis... | NA | 36363 | 219 | 219 | 97% | 1e-52 | 77.22 | 1317 | GU320726.1 |
| Lymphocystis disease virus 1 MCP gene for major capsid protein... | Lymphocystis... | NA | 36363 | 219 | 219 | 97% | 1e-52 | 77.22 | 1347 | AB212998.1 |
| Lymphocystis disease virus 2 LCDV-JP_Oita_2018 DNA, complete... | Lymphocystis... | NA | 159183 | 214 | 214 | 97% | 5e-51 | 76.87 | 186627 | LC534415.1 |
| Lymphocystis disease virus 1 strain PO6 major capsid protein... | Lymphocystis... | NA | 36363 | 214 | 214 | 97% | 5e-51 | 76.87 | 751 | KP184512.1 |
| Lymphocystis disease virus 1 isolate SA12 major capsid protein... | Lymphocystis... | NA | 36363 | 214 | 214 | 97% | 5e-51 | 76.87 | 1317 | GU320730.1 |
| Lymphocystis disease virus 1 isolate SSE11 major capsid protei... | Lymphocystis... | NA | 36363 | 214 | 214 | 97% | 5e-51 | 76.87 | 1296 | GU320729.1 |
| Lymphocystis disease virus from Paralichthys olivaceus major... | Lymphocystis... | NA | 36363 | 214 | 214 | 97% | 5e-51 | 76.87 | 1380 | AY849391.1 |
| Lymphocystis disease virus - isolate China, complete genome | Lymphocystis... | NA | 256729 | 214 | 214 | 97% | 5e-51 | 76.87 | 186250 | AY380826.1 |
| Lymphocystis disease virus 1 MCP gene for major capsid protein... | Lymphocystis... | NA | 36363 | 214 | 214 | 97% | 5e-51 | 76.87 | 1347 | AB212999.1 |
| Lymphocystis disease virus 1 MCP gene for major capsid protein... | Lymphocystis... | NA | 36363 | 214 | 214 | 97% | 5e-51 | 76.87 | 1347 | AB212997.1 |
| Lymphocystis disease virus strain KLDV-1 MCP gene, complete cds | Lymphocystis... | NA | 36363 | 214 | 214 | 97% | 5e-51 | 76.87 | 2521 | AY297741.1 |
| Lymphocystis disease virus major capsid protein (MCP) gene,... | Lymphocystis... | NA | 36363 | 214 | 214 | 97% | 5e-51 | 76.87 | 1380 | AY303804.1 |
| Lymphocystis disease virus major capsid protein gene, partial cds | Lymphocystis... | NA | 36363 | 214 | 214 | 97% | 5e-51 | 76.87 | 636 | AF126405.1 |
| Lymphocystis disease virus 1 MCP gene for major capsid protein... | Lymphocystis... | NA | 36363 | 210 | 210 | 97% | 6e-50 | 76.51 | 1347 | AB213000.1 |
| Lymphocystis disease virus strain Huangdao major capsid protei... | Lymphocystis... | NA | 358077 | 200 | 200 | 91% | 1e-46 | 76.81 | 348 | DQ279090.1 |
| Lymphocystis disease virus 1 strain SA64 major capsid protein... | Lymphocystis... | NA | 36363 | 175 | 175 | 70% | 5e-39 | 78.92 | 839 | KP184511.1 |
| Iridovirus Liz-CrIV genomic sequence | Iridovirus L... | NA | 2594309 | 93.3 | 93.3 | 73% | 1e-14 | 70.97 | 191409 | MN081869.1 |
| PREDICTED: Harpegnathos saltator uncharacterized LOC112588854... | Harpegnathos... | Jerdon's jum... | 610380 | 93.3 | 93.3 | 73% | 1e-14 | 70.97 | 1428 | XM_025300036.1 |
| Invertebrate iridescent virus Kaz2018, complete genome | Invertebrate... | NA | 2763244 | 88.7 | 88.7 | 73% | 5e-13 | 70.51 | 212482 | MT862761.1 |
| Chilo iridescent virus complete genome | Invertebrate... | NA | 176652 | 88.7 | 88.7 | 73% | 5e-13 | 70.51 | 212482 | AF303741.1 |
| Insect iridescent virus type 22 major structural protein (MSP)... | Simulium sp.... | NA | 10489 | 86.0 | 86.0 | 85% | 2e-12 | 68.27 | 2183 | M32799.1 |
| Tipoula iridescent virus type 1 capsid protein gene, complete cds | Tipula iride... | NA | 10490 | 86.0 | 86.0 | 85% | 2e-12 | 68.27 | 2461 | M33542.1 |
| Invertebrate iridescent virus 30 complete genome | Invertebrate... | NA | 345585 | 84.2 | 84.2 | 84% | 6e-12 | 67.61 | 198533 | HF920636.1 |
| Invertebrate iridescent virus 22 isolate IIV22Aberystwyth... | Invertebrate... | NA | 345198 | 84.2 | 84.2 | 84% | 6e-12 | 67.61 | 196456 | HF920634.1 |
| Invertebrate iridovirus 22 complete genome | Invertebrate... | NA | 1301279 | 84.2 | 84.2 | 84% | 6e-12 | 67.61 | 197693 | HF920633.1 |
| Costelytra zealandica iridescent virus major capsid protein... | Costelytra z... | NA | 68348 | 70.7 | 70.7 | 84% | 1e-07 | 66.40 | 1835 | AF025775.1 |
| Wiseana iridescent virus, complete genome | Wiseana irid... | NA | 68347 | 68.9 | 68.9 | 84% | 5e-07 | 66.26 | 205791 | GQ918152.1 |
| Wiseana iridescent virus major capsid protein gene, complete cds | Wiseana irid... | NA | 68347 | 68.9 | 68.9 | 84% | 5e-07 | 66.26 | 1700 | AF025774.1 |

| | | | | | | | | | | |
|---|---|---|---|---|---|---|---|---|---|---|
| Invertebrate iridovirus 25 complete genome | Invertebrate... | NA | 1301280 | 66.2 | 66.2 | 62% | 2e-06 | 68.09 | 204815 | HF920635.1 |
| Erythrocytic necrosis virus isolate SEQ_86 major capsid protei... | Erythrocytic... | NA | 1543320 | 61.7 | 61.7 | 36% | 7e-05 | 72.64 | 1326 | MK638677.1 |
| Erythrocytic necrosis virus isolate British Columbia 8 major... | Erythrocytic... | NA | 1543320 | 61.7 | 61.7 | 36% | 7e-05 | 72.64 | 1196 | KT211481.1 |
| Erythrocytic necrosis virus isolate Puget Sound 1 major capsid... | Erythrocytic... | NA | 1543320 | 61.7 | 61.7 | 36% | 7e-05 | 72.64 | 1211 | KT211480.1 |
| Lymphocystis disease virus 1 isolate LCDV-BRA major protein... | Lymphocystis... | NA | 36363 | 56.3 | 56.3 | 16% | 0.003 | 85.42 | 183 | KY909844.1 |
| Tinea semifulvella genome assembly, chromosome: 27 | Tinea semifu... | NA | 1101063 | 56.3 | 56.3 | 16% | 0.003 | 85.42 | 11809494 | OU342611.1 |
| Lymphocystis disease virus 1 isolate SSE21 major capsid protei... | Lymphocystis... | NA | 36363 | 56.3 | 56.3 | 18% | 0.003 | 83.93 | 606 | GU328641.1 |
| Mamestra brassicae genome assembly, chromosome: 28 | Mamestra bra... | cabbage moth | 55057 | 51.8 | 51.8 | 19% | 0.038 | 82.46 | 11281633 | LR991015.1 |

Alignments:

```
Query           1         TAAAATAACAGGAGAAGCTGTAGTATAATTAGA---TTGAACAGCTGCATGAGTTGTATT  57
MN128712.1      1         .................................---........................  57
L63545.1        71803     .................................---........................  71859
KT438164.1      292        .....C..T..T....AA..............---..........TG.......AC...  347
AB213004.1      957        .....C..T..T....AA..............---..........TG.......AC...  902
AB299164.1      953       ......T..G..T...CAC.....G........---...C..G...TG..A....ACG..  897
AY849392.1      971        .....C..T..T....AA..............---..........TG.......AC...  916
AY823414.1      971        .....C..T..T....AA..............---..........TG.......AC...  916
GU939626.2      958       .........G.......AA..T...........---...T..C...TG...C...ACG..  902
GU290550.1      939       ..T......G.......AA..T...........---...T..C...TG..AC...AC...  883
AB247938.1      957        ..G..T..........AAC.G........G..---...T..C...TG......AACG..  902
NC_055603.1     34033        ...G..G..G....AAC.G........G..---...T......TG......AAC...  33980
EF059992.1      957        ..G..T..G.......AAC.G........G..---...T..C...TG......AACG..  902
EF378607.1      971        ..G..T..G.......AAC.G........G..---...T..C...TG......AACG..  916
EF103188.1      971        ..G..T..G.......AAC.G........G..---...T..C...TG......AACG..  916
MK250973.1      936                             ........G..---...T......TG......AAC...  902
AB299163.1      952        .....G..C.......AAC.G........G..---...T......TG.......AC...  897
GU320734.1      938        .....G..C.......AAC.G........G..---...T..G...TG.......ACG..  883
GU320731.1      836        .....G..C.......AAC.G........G..---...T..G...TG.......ACG..  781
GU320724.1      938        .....G..C.......AAC.G........G..---...T..G...TG.......ACG..  883
EF184306.1      938        .....G..C.......AAC.G........G..---...T..G...TG.......ACG..  883
HE650105.1      938        .....G..C.....G.AAC.G...........---...C..G...TG.......ACG..  883
GU320739.1      938        .....G..C.....G.AAC.G...........---...C..G...TG.......ACG..  883
GU320738.1      761        .....G..C.....G.AAC.G...........---...C..G...TG.......ACG..  706
GU320737.1      938        .....G..C.....G.AAC.G...........---...C..G...TG.......ACG..  883
GU320735.1      938        .....G..C.....G.AAC.G........G..---...T..G...TG.......ACG..  883
KJ408271.1      920        ........C..G....AAC.G........G..---...T..G...TG.......ACG..  865
KX643370.1      65916      .....G..C.....G.AAC.G........G..---...T..G...TG.......ACG..  65861
GU320736.1      938        .....G..C.....G.AAC.G........G..---...T..G...TG.......ACG..  883
GU320732.1      938        .....G..C.....G.AAC.G........G..---...T..G...TG.......ACG..  883
GU320725.1      938        .....G..C.....G.AAC.G........G..---...T..G...TG.......ACG..  883
GU320727.1      917        .....G..C.....G.AAC.G........G..---...T..G...TG.......ACG..  862
GU320726.1      938        .....G..C.....G.AAC.G........G..---...T..G...TG.......ACG..  883
AB212998.1      952        .....T..........AAC.G..G.....G..---...T.T....TG..AG..AAC...  897
LC534415.1      31859      ..G..T..........AAC.G..G.....G..---...T.T....TG..AG..AAC...  31914
KP184512.1      372        ..G..T..........AAC.G..G.....G..---...T.T....TG..AG..AAC...  317
GU320730.1      938        .....G..C.....G.AAC.G........G..---...T..G...TG.......ACG..  883
GU320729.1      917        .....G..C.....G.AAC.G........G..---...T..G...TG.......ACG..  862
AY849391.1      971        ..G..T..........AAC.G..G.....G..---...T.T....TG..AG..AAC...  916
AY380826.1      31813      ..G..T..........AAC.G..G.....G..---...T.T....TG..AG..AAC...  31868
AB212999.1      952        ..G..T..........AAC.G..G.....G..---...T.T....TG..AG..AAC...  897
AB212997.1      952        ..G..T..........AAC.G..G.....G..---...T.T....TG..AG..AAC...  897
AY297741.1      1009       ..G..T..........AAC.G..G.....G..---...T.T....TG..AG..AAC...  954
AY303804.1      971        ..G..T..........AAC.G..G.....G..---...T.T....TG..AG..AAC...  916
AF126405.1      515        ..G..T..........AAC.G..G.....G..---...T.T....TG..AG..AAC...  460
AB213000.1      952        ..G..T..........AAC.G..G.....G..---...T.T....TG..AG..AAC...  897
DQ279090.1      265        ..G..T..........AAC.G..G.....G..---...T.T....TG..AG..AAC...  210
MN081869.1      118005       ...............G..T.....G..T..AGG....TT..AA--.A.-........  118058
XM_025300036.1  993          ...............G..T.....G..T..AGG....TT..AA--.A.-........  940
MT862761.1      129166       ...............G..T.....G..T..AGG....TT..AA--.A.-........  129219
AF303741.1      129166       ...............G..T.....G..T..AGG....TT..AA--.A.-........  129219
M32799.1        1635         ..C...C.......AA....C......T..---CCATT.T......GAT...T....  1582
M33542.1        1581         ..C...T.......AA....C......T..---CCATT.T......GAT...T....  1528
HF920636.1      81775           .........T.AG....C......T..---CCATT...AA..CCC...CT....  81725
HF920634.1      79369           .........T.AG....C......T..---CCATT...AA...CC...CT....  79319
HF920633.1      80789           .........T.AG....C......T..---CCATT...AA..CCC...CT....  80739
AF025775.1      1080            ...........A...T.C...G..T..---CCATT.C....A.CT...CT.G..  1030
```

```
GQ918152.1      9211            ..C.......A...T.C...G..T..---CCACT...AC...CT....T....  9162
AF025774.1      1203            ..C.......A...T.C...G..T..---CCACT...AC...CT....T....  1154
MK638677.1      993         ...T................CC.......---CCAC..GTT..GG.A...GA....  940
KT211481.1      1038        ...T................CC.......---CCAC..GTT..GG.A...GA....  985
KT211480.1      1038        ...T................CC.......---CCAC..GTT..GG.A...GA....  985
LR991015.1      1199433                                                         ....  1199430

Query           58          TCTTACACCAAAAAATAACAA---TTTAATAGCGTGTGAAAACCGAATATCAAAATTAGG  114
MN128712.1      58          .....................---....................................  114
L63545.1        71860       .....................---....................................  71916
KT438164.1      348         A.GC..........C..A.T---.........A..A..G..T.....G.....G.....  404
AB213004.1      901         A.GC..........C..A.T---.........A..A..G..T.....G.....G.....  845
AB299164.1      896         G.G...G..........A.T---...........A.....T.T..............  840
AY849392.1      915         A.GC..........C..A.T---.........A..A..G..T.....G.....G.....  859
AY823414.1      915         A.GC.....G....C..A.T---.........A..A..G..T.....G.....G.....  859
GU939626.2      901         ...............C.GAGT---...........C.....T..G.....G........  845
GU290550.1      882         ..G...T..........A.G---C..........A..G..T..........G.....  826
AB247938.1      901         G.G......G.....A.GG.T---............A.....T..G..G..........  845
NC_055603.1     33979       G.G.........G..A.GAGT---............A..G..T..T......T.......  33923
EF059992.1      901         A.G............A.GA.C---............A..G..T..G..G..........  845
EF378607.1      915         A.G............A.GA.C---............A..G..T..G..G..........  859
EF103188.1      915         A.G............A.GA.C---............A..G..T..G..G..........  859
MK250973.1      901         G.G.........G..A.GAGT---............A..G..T..T......T.......  845
AB299163.1      896         G.G......G.....A.GAGT---......G..A..A.....T.T...G...T.......  840
GU320734.1      882         G.G............A.GA.T---...G..G.....A..G..T.....G..GT.......  826
GU320731.1      780         G.G............A.GA.T---...G..G.....A..G..T.....G..GT.......  724
GU320724.1      882         G.G............A.GA.T---...G..G.....A..G..T.....G..GT.......  826
EF184306.1      882         G.G............A.GA.T---...G..G.....A..G..T.....G..GT.......  826
HE650105.1      882         G.G............A.GA.T---...G..G.....A..G..T.....G..GT.......  826
GU320739.1      882         G.G............A.GA.T---...G..G.....A..G..T.....G..GT.......  826
GU320738.1      705         G.G............A.GA.T---...G..G.....A..G..T.....G..GT.......  649
GU320737.1      882         G.G............A.GA.T---...G..G.....A..G..T.....G..GT.......  826
GU320735.1      882         G.G............A.GA.T---...G..G.....A..G..T.....G..GT.......  826
KJ408271.1      864         G.G...G..G.....A.GAGT---...G..G.....A.....T.TG..G..GT....G..  808
KX643370.1      65860       G.G............A.GA.T---...G..G.....A..G..T.....G..GT.......  65804
GU320736.1      882         G.G............A.GA.T---...G..G.....A..G..T.....G..GT.......  826
GU320732.1      882         G.G............A.GA.T---...G..G.....A..G..T.....G..GT.......  826
GU320725.1      882         G.G............A.GA.T---...G..G.....A..G..T.....G..GT.......  826
GU320727.1      861         G.G............A.GA.T---...G..G.....A..G..T.....G..GT.......  805
GU320726.1      882         G.G............A.GA.T---...G..G.....A..G..T.....G..GT.......  826
AB212998.1      896         A.GC.....G.....A.GG.T---......G..A..A.....T.TG..G..........  840
LC534415.1      31915       A.GC.....G.....A.GG.T---......G..A..A.....T.TG..G..........  31971
KP184512.1      316         A.GC.....G.....A.GG.T---......G..A..A.....T.TG..G..........  260
GU320730.1      882         G.G............A.GA.T---...G..G.....A..G..T.....G..GT....G..  826
GU320729.1      861         G.G............A.GA.T---...G..G.....A..G..T.....G..GT....G..  805
AY849391.1      915         A.GC.....G.....A.GG.T---......G..A..A.....T.TG..G..........  859
AY380826.1      31869       A.GC.....G.....A.GG.T---......G..A..A.....T.TG..G..........  31925
AB212999.1      896         A.GC.....G.....A.GG.T---......G..A..A.....T.TG..G..........  840
AB212997.1      896         A.GC.....G.....A.GG.T---......G..A..A.....T.TG..G..........  840
AY297741.1      953         A.GC.....G.....A.GG.T---......G..A..A.....T.TG..G..........  897
AY303804.1      915         A.GC.....G.....A.GG.T---......G..A..A.....T.TG..G..........  859
AF126405.1      459         A.GC.....G.....A.GG.T---......G..A..A.....T.TG..G..........  403
AB213000.1      896         A.GC.....G.....A.GG.T---......G..A..A.....T.TG..G..........  840
DQ279090.1      209         A.GC.....G.....A.GG.T---......G..A..A.....T.TG..G..........  153
KP184511.1      838                                    ...G..G.....A..G..T.....G..GT.......  803
MN081869.1      118059      ...A...G....G..A.GTGC---.........A..A..G..G........GT.G.....  118115
XM_025300036.1  939         ...A...G....G..A.GTGC---.........A..A..G..G........GT.G.....  883
MT862761.1      129220      ...A...G....G..A.GTGC---.........A..A..G..G........GT.G.....  129276
AF303741.1      129220      ...A...G....G..A.GTGC---.........A..A..G..G........GT.G.....  129276
M32799.1        1581        ..G....G....G.....TGC---...G..T..A..A..G..A...........TG.T..  1525
M33542.1        1527        ..G....G....G.....TGC---...G..T..A..A..G..A...........TG.T..  1471
HF920636.1      81724       A.GA..TGA...G.....AGC---...G..G..A..........T..........G....  81668
HF920634.1      79318       A.GA..TGA...G.....AGC---...G..G..A..........T..........G....  79262
HF920633.1      80738       A.GA..TGA...G.....AGC---...G..G..A..........T..........G....  80682
AF025775.1      1029        ...A..TGA...G..A.GAGC---......G.....A..G....T.........TG.T..  973
GQ918152.1      9161        A.GA..TGA...G..C..AGC---......G.....G.......T......G..TG.T..  9105
AF025774.1      1153        A.GA..TGA...G..C..AGC---......G.....G.......T......G..TG.T..  1097
HF920635.1      83775                   ....G.....AGC......G..A..A.......T......G..TG.T..  83727
```

```
MK638677.1      939       ......T.....T....GAGC---C..G.C...A..A.....T.T..C.......      888
KT211481.1      984       ......T.....T....GAGC---C..G.C...A..A.....T.T..C.......      933
KT211480.1      984       ......T.....T....GAGC---C..G.C...A..A.....T.T..C.......      933
OU342611.1      10927161         .....T..GT....---.....A............AA..T...........  10927114
LR991015.1      1199429   ...-....A..T......-..---.....A..T....T...T..TT..........     1199379

Query           115       -GCTTGGAATAGTTAACGGTTGAAATACATGTT---T----T----GGCGCAGTTTGAAC  162
MN128712.1      115       -................................---.----.----..............  162
L63545.1        71917     -................................---.----.----..............  71964
KT438164.1      405       -...A...........T................---.----A----..T..T.....T..  452
AB213004.1      844       -...A...........T................---.----A----..T..T.....T..  797
AB299164.1      839       -A..A...........A................---.----C----.....T.....T..  792
AY849392.1      858       -...A...........T................---.----A----..T..T.....T..  811
AY823414.1      858       -...A...........T................---.----A----..T..T.....T..  811
GU939626.2      844       -...A...........A...........G....---.----.----..A..G.....T..  797
GU290550.1      825       -A..C..G..T.....A...........G....---.----.----..A........T..  778
AB247938.1      844       -...A...........A..............C.---.----A----.....T.....T..  797
NC_055603.1     33922     -A........G.....A................---.----.----..A..C.....T..  33875
EF059992.1      844       -...A...........A..............C.---.----G----.....T.....T..  797
EF378607.1      858       -...A...........A..............C.---.----G----.....T.....T..  811
EF103188.1      858       -...A...........A..............C.---.----G----.....T.....T..  811
MK250973.1      844       -A........G.....A................---.----.----..A..C.....TG.  797
AB299163.1      839       -A..A........C..T........C.......---.----.----..T..T.....T..  792
GU320734.1      825       -A..G..G........A...........G....---.----A----..T..T..C..T..  778
GU320731.1      723       -A..G..G........A...........G....---.----A----..T..T..C..T..  676
GU320724.1      825       -A..G..G........A...........G....---.----A----..T..T..C..T..  778
EF184306.1      825       -A..G..G........A...........G....---.----A----..T..T..C..T..  778
HE650105.1      825       -A..G..G........A...........G....---.----A----..T..T..C..T..  778
GU320739.1      825       -A..G..G........A...........G....---.----A----..T..T..C..T..  778
GU320738.1      648       -A..G..G........A...........G....---.----A----..T..T..C..T..  601
GU320737.1      825       -A..G..G........A...........G....---.----A----..T..T..C..T..  778
GU320735.1      825       -A..G..G........A...........G....---.----A----..T..T..C..T..  778
KJ408271.1      807       -A........G.....A...........G....---.----A----.....C.....T..  760
KX643370.1      65803     -A..G..G........A...........G....---.----A----..T..T..C..T..  65756
GU320736.1      825       -A..G..G........A...........G....---.----A----..T..T..C..T..  778
GU320732.1      825       -A..G..G........A...........G....---.----A----..T..T..C..T..  778
GU320725.1      825       -A..G..G........A...........G....---.----A----..T..T..C..T..  778
GU320727.1      804       -A..G..G........A...........G....---.----A----..T..T..C..T..  757
GU320726.1      825       -A..G..G........A...........G....---.----A----..T..T..C..T..  778
AB212998.1      839       -A.............GA................---.----A----..T..T.....T..  792
LC534415.1      31972     -A.............GA................---.----A----..T..T.....T..  32019
KP184512.1      259       -A.............GA................---.----A----..T..T.....T..  212
GU320730.1      825       -A..G..G........A...........G....---.----A----..T..T..C..T..  778
GU320729.1      804       -A..G..G........A...........G....---.----A----..T..T..C..T..  757
AY849391.1      858       -A.............GA................---.----A----..T..T.....T..  811
AY380826.1      31926     -A.............GA................---.----A----..T..T.....T..  31973
AB212999.1      839       -A.............GA................---.----A----..T..T.....T..  792
AB212997.1      839       -A.............GA................---.----A----..T..T.....T..  792
AY297741.1      896       -A.............GA................---.----A----..T..T.....T..  849
AY303804.1      858       -A.............GA................---.----A----..T..T.....T..  811
AF126405.1      402       -A.............GA................---.----A----..T..T.....T..  355
AB213000.1      839       -A.............GA................---.----A----..T..T.....T..  792
DQ279090.1      152       -A.............GA....C...........---.----A----..T..T.....T..  105
KP184511.1      802       -A..G..G........A...........G....---.----A----..T..T..C..T..  755
MN081869.1      118116    A..A.-C.T......TT..A.TCC.A.....AC---G----.----..A.....C.....  118163
XM_025300036.1  882       A..A.C-.T......TT..A.TCC.A.....AC---G----.----..A.....C.....  835
MT862761.1      129277    A..A.-C.T.....TTT..A.TCC.A.....AC---G----.----..A.....C.....  129324
AF303741.1      129277    A..A.-C.T.....GTT..A.TCC.A.....AC---G----.----..A.....C.....  129324
M32799.1        1524      -A..A.C.T.G..C..A...--.C...-..T..GACG----C----.....C........  1477
M33542.1        1470      -A..A.C.T.G..C..A...--.C...-..T..---GACG-.----..T..C........  1423
HF920636.1      81667     -A....C.T.T..A.GA..----.G..T.AT..---.GACG.----..T...........  81620
HF920634.1      79261     -A....C.T.T..A.GA..----.G..T.AT..---.GACG.----..T...........  79214
HF920633.1      80681     -A....C.T.T..A.GA..----.G..T.AT..---.GACG.----..T...........  80634
AF025775.1      972       -CA...C.T.G..C..A..----.G..T.GT..---.GACG.----..T...........  925
GQ918152.1      9104      -A..C.C.T....C..A..----.G..T.AT..---.----GACGC...........T..  9057
AF025774.1      1096      -A..C.C.T....C..A..----.G..T.AT..---.----GACGC...........T..  1049
HF920635.1      83726     -.....C.T....C..A..----.G..T.AT..---.----GACGC..T........T..  83679
```

```
Query           163       TTGTTCCACTAATATATCTCGAGGAGTTGTACCCATAAGTCGCCGTTCTTCATTTGTAAC  222
MN128712.1      163       ............................................................  222
L63545.1        71965     ............................................................  72024
KT438164.1      453       ......T....G........T...TACA........C.A...A...........A..T..  512
AB213004.1      796       ......T....G........T...TACA........C.A...A...........A..T..  737
AB299164.1      791       ......T.................TACG........T.A..TA...........G..T..  732
AY849392.1      810       ......T....G........T...TACA........C.A...A..........CA..T..  751
AY823414.1      810       ......T....G........T...TACA........C.A...A...........A..T..  751
GU939626.2      796       ......G.....A.......T...C..AA.......C.A...A........G..A..T..  737
GU290550.1      777       ......T..C..A.......T...TACA........T.....G........G..G..T..  718
AB247938.1      796       ......T..C..A.......TG..TAC.........C.A...A........G..A..T..  737
NC_055603.1     33874     C.....T.TC..A.......TG..TA.G........C.A...............G..C..  33815
EF059992.1      796       ......T..C..A.......TG..TAC.........C.A...A........G..A..T..  737
EF378607.1      810       ......T..C..A.......TG..TAC.........C.A...A........G..A..T..  751
EF103188.1      810       ......T..C..A.......TG..TAC.........C.A...A........G..A..T..  751
MK250973.1      796       C.....T.TC..A.......TG..TA.G........C.A...............G..C..  737
AB299163.1      791       ......T..C..A.......TG..TACC........C.A...A...........A..C..  732
GU320734.1      777       ...C..T..C..G.......TG..TACC........C.A...A........G..G..C..  718
GU320731.1      675       ...C..T..C..G.......TG..TACC........C.A...A........G..G..C..  616
GU320724.1      777       ...C..T..C..G.......TG..TACC........C.A...A........G..G..C..  718
EF184306.1      777       ...C..T..C..G.......TG..TACC........C.A...A........G..G..T..  718
HE650105.1      777       ...C..T..C..G.......TG..TACC........C.A...A........G..A..T..  718
GU320739.1      777       ...C..T..C..G.......TG..TACC........C.A...A........G..A..T..  718
GU320738.1      600       ...C..T..C..G.......TG..TACC........C.A...A........G..A..T..  541
GU320737.1      777       ...C..T..C..G.......TG..TACC........C.A...A........G..A..T..  718
GU320735.1      777       ...C..T..C..G.......TG..TACC........C.A...A........G..G..T..  718
KJ408271.1      759       ...C..T.....A.......T...TA.C........C.A...A........G..G..C..  700
KX643370.1      65755     ...C..T..C..G.......TG..TACC........C.A...A........G..G..T..  65696
GU320736.1      777       ...C..T..C..G.......TG..TACC........C.A...A........G..G..T..  718
GU320732.1      777       ...C..T..C..G.......TG..TACC........C.A...A........G..G..T..  718
GU320725.1      777       ...C..T..C..G.......TG..TACC........C.A...A........G..G..T..  718
GU320727.1      756       ...C..T..C..G.......TG..TACC........C.A...A........G..G..T..  697
GU320726.1      777       ...C..T..C..G.......TG..TACC........C.A...A........G..G..T..  718
AB212998.1      791       C.....T..C..G..G....T...TAC.........T.AA..A.....C.....G..T..  732
LC534415.1      32020     C.....T..C..G..G....T...TAC.........T.AA..A.....C.....G..T..  32079
KP184512.1      211       C.....T..C..G..G....T...TAC.........T.AA..A.....C.....G..T..  152
GU320730.1      777       ...C..T..C..G.......TG..TACC........C.A...A........G..G..T..  718
GU320729.1      756       ...C..T..C..G.......TG..TACC........C.A...A........G..G..T..  697
AY849391.1      810       C.....T..C..G..G....T...TAC.........T.AA..A.....C.....G..T..  751
AY380826.1      31974     C.....T..C..G..G....T...TAC.........T.AA..A.....C.....G..T..  32033
AB212999.1      791       C.....T..C..G..G....T...TAC.........T.AA..A.....C.....G..T..  732
AB212997.1      791       C.....T..C..G..G....T...TAC.........T.AA..A.....C.....G..T..  732
AY297741.1      848       C.....T..C..G..G....T...TAC.........T.AA..A.....C.....G..T..  789
AY303804.1      810       C.....T..C..G..G....T...TAC.........T.AA..A.....C.....G..T..  751
AF126405.1      354       C.....T..C..G..G....T...TAC.........T.AA..A.....C.....G..T..  295
AB213000.1      791       C.....T..C..G..G....T...TAC.........T.AA..A.....C.....G..T..  732
DQ279090.1      104       C.....T..C..G..G....T...TAC.........T.AA..A.....C.....G..T..  45
KP184511.1      754       ...C..T..C..G.......TG..TACC........C.A...A........G..A..T..  695
MN081869.1      118164    ......A..C..A.......TTAC..AGCAT......C...TT..C........        118217
XM_025300036.1  834       ......A..C..A.......TTAC..AGCAT......C...TT..C........        781
MT862761.1      129325    ......A..C..A.......TTAC..AGCAT......C...TT..C........        129378
AF303741.1      129325    ......A..C..A.......TTAC..AGCAT......C...TT..C........        129378
M32799.1        1476      C.....A.TC..A..G......ATG.CACA......TCTA..A........G..G.AG..  1417
M33542.1        1422      C.....A.TC..A..G......ATG.CACA......TCTA..A........G..G.AG..  1363
HF920636.1      81619     ......A.T..GG..G....T.AT..CACA......TCTA..A...........AC.T..  81560
HF920634.1      79213     ......A.T..GG..G....T.AT..CACA......TCTA..A...........AC.T..  79154
HF920633.1      80633     ......A.T...G..G....T.AT..CACA......TCTA..A...........AC.T..  80574
AF025775.1      924       ......A.TC..A.......T.ATG.CACA......CCT..TT........G..G.A...  865
GQ918152.1      9056      C.....A.TC..A..G......AT..CACA......TCTA..A........G..AC.T..  8997
AF025774.1      1048      C.....A.TC..A..G......AT..CACA......TCTA..A........G..AC.T..  989
HF920635.1      83678     C.....A.TC..A..G......AT..CACA......TCTA..A........G..AC.T..  83619

Query           223       CACTGCATTAGTAATCCATACTTGAACGTCTTTTAAATCAGGTTTT--CCATATTCCAAA  280
MN128712.1      223       ..............................................--............  280
L63545.1        72025     ..............................................--........T...  72082
KT438164.1      513       T...A....G..............T..A..................--..CC.AA.T...  570
AB213004.1      736       T...A....G..............T..A..................--..CC.AA.T...  679
AB299164.1      731       T...A.G.................T..A..................--..TCC.A.T...  674
```

```
AY849392.1      750       T...A....G.............T..A.................--..CC.AA.T...  693
AY823414.1      750       T...A....G.............T..A.................--..            703
GU939626.2      736       G...A.G.....................GG...G...G......--..            689
GU290550.1      717       A...A.G...................A......G...G......CC...C...--...  660
AB247938.1      736       T...A.G..G.............T..A..C..C...........A--..CC....T...  679
NC_055603.1     33814     A...A....G........C.....T..A..C.............A--..CC....T...  33757
EF059992.1      736       T...A.G..G.............T..A..C..C...........A--..CC....T...  679
EF378607.1      750       T...A.G..G.............T..A..C..C...........A--..CC....T...  693
EF103188.1      750       T...A.G..G.............T..A..C..C...........A--..CC....T...  693
MK250973.1      736       A...A....G........C.....T..A..C.............A--..CC....T...  679
AB299163.1      731       T..AA....G..G..........T..A..C..............A--..CC.C..T...  674
GU320734.1      717       ...CA.G................C....................A--..CC....T...  660
GU320731.1      615       ...CA.G................C....................A--..CC....T...  558
GU320724.1      717       ...CA.G................C....................A--..CC....T...  660
EF184306.1      717       ....A.G.....G..........C....................A--..CC....T...  660
HE650105.1      717       ...CA.G.....G..........C....................A--..CC....T...  660
GU320739.1      717       ...CA.G.....G..........C....................A--..CC....T...  660
GU320738.1      540       ...CA.G.....G..........C....................A--..CC....T...  483
GU320737.1      717       ...CA.G.....G..........C....................A--..CC....T...  660
GU320735.1      717       ...CA.G................C....................A--..CC....T...  660
KJ408271.1      699       ...AA.G.....G..........T..A...........G.....A--..CC....T...  642
KX643370.1      65695     ...CA.G.....G..........C....................A--..CC....T...  65638
GU320736.1      717       ...CA.G.....G..........C....................A--..CC....T...  660
GU320732.1      717       ...CA.G.....G..........C....................A--..CC....T...  660
GU320725.1      717       ...CA.G.....G..........C....................A--..CC....T...  660
GU320727.1      696       ...CA.G.....G..........C....................A--..CC....T...  639
GU320726.1      717       ...CA.G.....G..........C....................A--..CC....T...  660
AB212998.1      731       T...A..................C..A..C..............A--..CC.G..T...  674
LC534415.1      32080     T...A..................C..A..C..............A--..CC.G..T...  32137
KP184512.1      151       T...A..................C..A..C..............A--..CC.G..T...  94
GU320730.1      717       ...CA.G.....G..........C....................A--..CC....T...  660
GU320729.1      696       ...CA.G.....G..........C....................A--..CC....T...  639
AY849391.1      750       T...A..................C..A..C..............A--..CC.G..T...  693
AY380826.1      32034     T...A..................C..A..C..............A--..CC.G..T...  32091
AB212999.1      731       T...A..................C..A..C..............A--..CC.G..T...  674
AB212997.1      731       T...A..................C..A..C..............A--..CC.G..T...  674
AY297741.1      788       T...A..................C..A..C..............A--..CC.G..T...  731
AY303804.1      750       T...A..................C..A..C..............A--..CC.G..T...  693
AF126405.1      294       T...A..................C..A..C..............A--..CC.G..T...  237
AB213000.1      731       T...A..................C..A..C...........C..A--..CC.G..T...  674
DQ279090.1      44        T...A..................C..A..C...........                    3
KP184511.1      694       ...CA.G.....G..........C....................A--..CC....T...  637
M32799.1        1416      G.TG....AGT..GC...........                                   1390
M33542.1        1362      G.TG....AGT..GC...........                                   1336
HF920636.1      81559     A.TA....AGT..GC...........                                   81533
HF920634.1      79153     A.TA....AGT.GGC...........                                   79127
HF920633.1      80573     A.TA....AGT.GGC...........                                   80547
AF025775.1      864       A.TG..G.A.T.TGC...........                                   838
GQ918152.1      8996      A.TG..G.A.T..GC...........                                   8970
AF025774.1      988       A.TG..G.A.T..GC...........                                   962
HF920635.1      83618     A.TG..G.A.T..GC...........                                   83592
KY909844.1      183                   ...........T.....C........G.....A--..CC....T...  138
GU328641.1      606                   ...........C.........G...G......CC...C...--...  561

Query           281       TCAGCAGC  288
MN128712.1      281       ........  288
L63545.1        72083     ........  72090
KT438164.1      571       ..TC....  578
AB213004.1      678       ..TC....  671
AB299164.1      673       ...       671
AY849392.1      692       ..TC....  685
GU290550.1      659       .....G..  652
AB247938.1      678       ...       676
NC_055603.1     33756     ...       33754
EF059992.1      678       ...       676
EF378607.1      692       ...       690
EF103188.1      692       ...       690
MK250973.1      678       ...       676
```

| | | | |
|---|---|---|---|
| AB299163.1 | 673 | ... | 671 |
| GU320734.1 | 659 | .. | 658 |
| GU320731.1 | 557 | .. | 556 |
| GU320724.1 | 659 | .. | 658 |
| EF184306.1 | 659 | .. | 658 |
| HE650105.1 | 659 | .. | 658 |
| GU320739.1 | 659 | .. | 658 |
| GU320738.1 | 482 | .. | 481 |
| GU320737.1 | 659 | .. | 658 |
| GU320735.1 | 659 | .. | 658 |
| KJ408271.1 | 641 | ... | 639 |
| KX643370.1 | 65637 | .. | 65636 |
| GU320736.1 | 659 | .. | 658 |
| GU320732.1 | 659 | .. | 658 |
| GU320725.1 | 659 | .. | 658 |
| GU320727.1 | 638 | .. | 637 |
| GU320726.1 | 659 | .. | 658 |
| AB212998.1 | 673 | .. | 672 |
| LC534415.1 | 32138 | .. | 32139 |
| KP184512.1 | 93 | .. | 92 |
| GU320730.1 | 659 | .. | 658 |
| GU320729.1 | 638 | .. | 637 |
| AY849391.1 | 692 | .. | 691 |
| AY380826.1 | 32092 | .. | 32093 |
| AB212999.1 | 673 | .. | 672 |
| AB212997.1 | 673 | .. | 672 |
| AY297741.1 | 730 | .. | 729 |
| AY303804.1 | 692 | .. | 691 |
| AF126405.1 | 236 | .. | 235 |
| AB213000.1 | 673 | .. | 672 |
| KP184511.1 | 636 | .. | 635 |
| KY909844.1 | 137 | .. | 136 |
| GU328641.1 | 560 | .....G.. | 553 |
