# Supplementary material for: Initial Evidence That Gilthead Seabream (Sparus aurata L.) Is a Host for Lymphocystis Disease Virus Genotype I
Source: Animals (Basel). 2021 Oct 22;11(11):3032. doi: 10.3390/ani11113032 (PMC8614504; doi:10.3390/ani11113032)
Supplement: Supplementary file 1 [file animals-11-03032-s001.zip › animals-1331593-Supplementary Table S1 Tabulated BLASTn alignment results..pdf]

blastn
Iteration: 0
Query:
RID: M4KSYF2201R
Database: nt
67 hits found
Fields:

query acc.ver	subject acc.ver	% identity	alignment length	mismatches	gap opens	q. start	q. end	s. start	s. end	E value	bit score
Query_379521	MN128712.1	100.000	288	0	0	1	288	288	1	2.39e-143	520
Query_379521	L63545.1	99.653	288	1	0	1	288	72090	71803	1.02e-141	516
Query_379521	KT438164.1	82.230	287	51	0	1	287	578	292	2.56e-73	288
Query_379521	AB213004.1	82.230	287	51	0	1	287	671	957	2.56e-73	288
Query_379521	AB299164.1	82.332	283	50	0	6	288	671	953	8.94e-73	286
Query_379521	AY849392.1	81.882	287	52	0	1	287	685	971	3.12e-72	284
Query_379521	AY823414.1	83.271	269	45	0	19	287	703	971	1.09e-71	283
Query_379521	GU939626.2	82.963	270	46	0	19	288	689	958	3.80e-71	280
Query_379521	GU290550.1	81.034	290	51	2	1	288	652	939	6.87e-68	270
Query_379521	AB247938.1	79.433	282	58	0	6	287	676	957	2.25e-61	248
Query_379521	NC_055603.1	79.286	280	58	0	6	285	33754	34033	2.74e-60	244
Query_379521	EF059992.1	79.078	282	59	0	6	287	676	957	9.55e-60	243
Query_379521	EF378607.1	79.078	282	59	0	6	287	690	971	9.55e-60	243
Query_379521	EF103188.1	79.078	282	59	0	6	287	690	971	9.55e-60	243
Query_379521	MK250973.1	80.077	261	52	0	6	266	676	936	4.06e-58	237
Query_379521	AB299163.1	78.014	282	62	0	6	287	671	952	6.03e-56	230
Query_379521	GU320734.1	77.936	281	62	0	7	287	658	938	2.10e-55	228
Query_379521	GU320731.1	77.936	281	62	0	7	287	556	836	2.10e-55	228
Query_379521	GU320724.1	77.936	281	62	0	7	287	658	938	2.10e-55	228
Query_379521	EF184306.1	77.936	281	62	0	7	287	658	938	2.10e-55	228
Query_379521	HE650105.1	77.580	281	63	0	7	287	658	938	8.94e-54	224
Query_379521	GU320739.1	77.580	281	63	0	7	287	658	938	8.94e-54	224
Query_379521	GU320738.1	77.580	281	63	0	7	287	481	761	8.94e-54	224
Query_379521	GU320737.1	77.580	281	63	0	7	287	658	938	8.94e-54	224
Query_379521	GU320735.1	77.580	281	63	0	7	287	658	938	8.94e-54	224
Query_379521	KJ408271.1	77.305	282	64	0	6	287	639	920	3.12e-53	221
Query_379521	KX643370.1	77.224	281	64	0	7	287	65636	65916	1.09e-52	219
Query_379521	GU320736.1	77.224	281	64	0	7	287	658	938	1.09e-52	219
Query_379521	GU320732.1	77.224	281	64	0	7	287	658	938	1.09e-52	219
Query_379521	GU320725.1	77.224	281	64	0	7	287	658	938	1.09e-52	219
Query_379521	GU320727.1	77.224	281	64	0	7	287	637	917	1.09e-52	219
Query_379521	GU320726.1	77.224	281	64	0	7	287	658	938	1.09e-52	219

Query_379521	AB212998.1	77.224	281	64	0	7	287	672	952	1.09e-52	219
Query_379521	LC534415.1	76.868	281	65	0	7	287	32139	31859	4.63e-51	214
Query_379521	KP184512.1	76.868	281	65	0	7	287	92	372	4.63e-51	214
Query_379521	GU320730.1	76.868	281	65	0	7	287	658	938	4.63e-51	214
Query_379521	GU320729.1	76.868	281	65	0	7	287	637	917	4.63e-51	214
Query_379521	AY849391.1	76.868	281	65	0	7	287	691	971	4.63e-51	214
Query_379521	AY380826.1	76.868	281	65	0	7	287	32093	31813	4.63e-51	214
Query_379521	AB212999.1	76.868	281	65	0	7	287	672	952	4.63e-51	214
Query_379521	AB212997.1	76.868	281	65	0	7	287	672	952	4.63e-51	214
Query_379521	AY297741.1	76.868	281	65	0	7	287	729	1009	4.63e-51	214
Query_379521	AY303804.1	76.868	281	65	0	7	287	691	971	4.63e-51	214
Query_379521	AF126405.1	76.868	281	65	0	7	287	235	515	4.63e-51	214
Query_379521	AB213000.1	76.512	281	66	0	7	287	672	952	5.64e-50	210
Query_379521	DQ279090.1	76.806	263	61	0	25	287	3	265	1.02e-46	200
Query_379521	KP184511.1	78.922	204	43	0	7	210	635	838	4.06e-39	175
Query_379521	MN081869.1	70.968	217	55	5	73	285	118217	118005	1.09e-14	93.3
Query_379521	XM_025300036.1	70.968	217	55	5	73	285	781	993	1.09e-14	93.3
Query_379521	MT862761.1	70.507	217	56	5	73	285	129378	129166	4.64e-13	88.7
Query_379521	AF303741.1	70.507	217	56	5	73	285	129378	129166	4.64e-13	88.7
Query_379521	M32799.1	68.273	249	73	3	40	285	1390	1635	1.62e-12	86.0
Query_379521	M33542.1	68.273	249	73	3	40	285	1336	1581	1.62e-12	86.0
Query_379521	HF920636.1	67.611	247	72	2	40	282	81533	81775	5.65e-12	84.2
Query_379521	HF920634.1	67.611	247	72	2	40	282	79127	79369	5.65e-12	84.2
Query_379521	HF920633.1	67.611	247	72	2	40	282	80547	80789	5.65e-12	84.2
Query_379521	AF025775.1	66.397	247	75	2	40	282	838	1080	1.25e-07	70.7
Query_379521	GQ918152.1	66.260	246	75	2	40	281	8970	9211	4.35e-07	68.9
Query_379521	AF025774.1	66.260	246	75	2	40	281	962	1203	4.35e-07	68.9
Query_379521	HF920635.1	68.085	188	49	3	40	220	83592	83775	1.52e-06	66.2
Query_379521	MK638677.1	72.642	106	29	0	180	285	888	993	6.45e-05	61.7
Query_379521	KT211481.1	72.642	106	29	0	180	285	933	1038	6.45e-05	61.7
Query_379521	KT211480.1	72.642	106	29	0	180	285	933	1038	6.45e-05	61.7
Query_379521	KY909844.1	85.417	48	7	0	7	54	136	183	0.003	56.3
Query_379521	OU342611.1	85.417	48	7	0	177	224	10927114	10927161	0.003	56.3
Query_379521	GU328641.1	83.929	56	5	2	1	54	553	606	0.003	56.3
Query_379521	LR991015.1	82.456	57	8	2	179	235	1199379	1199433	0.033	51.8
